# CD157 signaling promotes survival of acute myeloid leukemia cells and modulates sensitivity to cytarabine through regulation of anti-apoptotic Mcl-1

**DOI:** 10.1038/s41598-021-00733-5

**Published:** 2021-10-27

**Authors:** Yuliya Yakymiv, Stefania Augeri, Cristiano Bracci, Sara Marchisio, Semra Aydin, Stefano D’Ardia, Massimo Massaia, Enza Ferrero, Erika Ortolan, Ada Funaro

**Affiliations:** 1 Laboratory of Immunogenetics, Department of Medical Sciences, University of Torino, Via Santena 19, 10126 Torino, Italy; 2 Department of Oncology, Hematology, Immuno-Oncology and Rheumatology, University of Bonn, Bonn, Germany; 3 Division of Hematology, Department of Oncology, Presidio Molinette, AOU Città della Salute e della Scienza, Torino, Italy; 4 SC Ematologia, AO S. Croce e Carle, Cuneo, Italy

**Keywords:** Cancer, Biomarkers, Oncology

## Abstract

CD157/BST-1 (a member of the ADP-ribosyl cyclase family) is expressed at variable levels in 97% of patients with acute myeloid leukemia (AML), and is currently under investigation as a target for antibody-based immunotherapy. We used peripheral blood and bone marrow samples from patients with AML to analyse the impact of CD157-directed antibodies in AML survival and in response to cytarabine (AraC) ex vivo. The study was extended to the U937, THP1 and OCI-AML3 AML cell lines of which we engineered CD157-low versions by shRNA knockdown. CD157-targeting antibodies enhanced survival, decreased apoptosis and reduced AraC toxicity in AML blasts and cell lines. CD157 signaling activated the PI3K/AKT/mTOR and MAPK/ERK pathways and increased expression of Mcl-1 and Bcl-XL anti-apoptotic proteins, while decreasing expression of Bax pro-apoptotic protein, thus preventing Caspase-3 activation. The primary CD157-mediated anti-apoptotic mechanism was Bak sequestration by Mcl-1. Indeed, the Mcl-1-specific inhibitor S63845 restored apoptosis by disrupting the interaction of Mcl-1 with Bim and Bak and significantly increased AraC toxicity in CD157-high but not in CD157-low AML cells. This study provides a new role for CD157 in AML cell survival, and indicates a potential role of CD157 as a predictive marker of response to therapies exploiting Mcl-1 pharmacological inhibition.

## Introduction

Acute myeloid leukemia (AML) is a clinically and genetically heterogeneous malignant disease characterized by abnormal proliferation of undifferentiated myeloid progenitors in the bone marrow (BM) and peripheral blood, impaired hematopoiesis and an aggressive clinical course. Intensive remission-inducing chemotherapy, followed by consolidation chemotherapy or allogeneic hematopoietic stem cell transplantation, in eligible patients with a high risk of relapse, still remains the standard therapeutic paradigm. However, mortality remains high, and most patients succumb to their disease in less than 5 years^1,2^. The dismal prognosis of AML is largely due to the acquisition of resistance to chemotherapy and leukemia relapse, which often overlap with deregulation of the apoptotic machinery^3^. However, growing understanding of the molecular basis of AML have led to the introduction of new treatment options that exploit novel mechanisms of action^4^. New drugs are rapidly being developed to expand the AML treatment landscape, with the promise of ultimately improving patient outcomes, especially in elderly patients and relapsed/refractory AML, the areas of greatest unmet need^5^.

In AML, suppression of apoptosis by expression of anti-apoptotic members of the Bcl-2 family is one of the principal mechanisms underlying disease pathogenesis, resistance and relapse, making the anti-apoptotic members of the family attractive therapeutic targets^5,6^. The basic idea is to kill leukemic cells by targeting the pro-survival members of the Bcl-2 family proteins with specific small molecule inhibitors that mimic the function of the BH3-only proteins, hence named BH3-mimetics. Currently, six BH3-mimetic drugs have entered clinical trials^7,8^ whereas the Bcl-2 inhibitor, ABT-199/venetoclax has been approved for therapy^9,10^. Although highly active, venetoclax monotherapy has shown limited efficacy in AML patients, and it proved to be more effective when used in combination with either hypomethylating agents or low-dose AraC for treatment-naive elderly patients^11^. However, a non-negligible minority of patients are refractory^12^ while the majority of patients who achieved remission, ultimately relapse^13^. In a clinical trial on relapsed and refractory AML, sensitivity to venetoclax increased with increased Bcl-2 expression; in contrast, sensitivity decreased with increased expression of myeloid cell leukemia-1 (Mcl-1)^14^, a critical anti-apoptotic member of the Bcl-2 family and target of various inhibitors now being used in clinical trials, such as S63845. Therefore, on the one hand, AML treatment needs the rational design of effective drug combination strategies, on the other, we need to be able to identify patients most likely to benefit from therapies encompassing specific BH3-mimetic drugs, for example through the discovery of predictive biomarkers.

CD157/BST-1 glycoprotein was originally discovered as Mo5 myeloid antigen^15^ and subsequently identified by RF3 monoclonal antibody as a cell membrane protein expressed in bone marrow stromal cells where it supports the growth of hematopoietic B cell progenitors^16^, hence the name *b*one marrow *st*romal cell antigen *1* (BST-1). CD157 is expressed by normal granulocytes, monocytes and more immature myeloid precursors. CD157 interaction with the heparin binding domain of selected extracellular matrix (ECM) proteins (e.g., fibronectin, collagen type 1 and laminin) promotes intracellular signaling in leukocytes, and regulate their adhesion^17^ and transmigration^18^. As a GPI-anchored protein, CD157 must associate with β1 and β2-integrins for signaling to occur. CD157-directed agonistic antibodies are effective in mimicking the signaling effects of the myeloid cells/ECM interaction^19–21^.

We previously showed that in two non-hematological malignancies, ovarian carcinoma^22^ and mesothelioma^23^, CD157 overexpression correlated with tumor invasiveness, aggressiveness, and decreased sensitivity to platinum-based chemotherapy^24^, suggesting that CD157 is more than a bystander marker. In AML, CD157 is expressed both at diagnosis and relapse, with highest expression in myelomonocytic and monocytic AML subtypes (*e.g.*, M4 and M5 subtypes, according to FAB classification). In addition, CD157 is also expressed in CD34^+^CD38^−^ leukemia-initiating cells that contribute to AML relapse^25^. Moreover, CD157 is currently under investigation as a target for antibody-based immunotherapy in AML (NCT02353143)^26^. However, the functional role of CD157 in the biology of AML has never been explored. To address this issue, we used CD157-directed antibodies to reveal that CD157 activates pro-survival signals in AML and in response to treatment. The mechanistic basis of this pro-survival effect was investigated in detail using AML cell lines expressing high and low levels of CD157 that confirmed the correlation between high CD157 expression, apoptosis inhibition and reduced sensitivity to chemotherapy. High CD157-expressing cell lines showed increased activation of the PI3K/AKT/mTOR and MAPK/ERK signaling pathways, converging on Mcl-1. Our study reveals a functional role of CD157 in pro-survival signaling in AML cells and provides a rationale for the use of CD157 as a predictive marker of response to therapies exploiting Mcl-1 pharmacological inhibition.

## Materials and methods

### Patients

Peripheral blood (n = 11) or bone marrow (n = 8) samples from patients with newly diagnosed AML without any prior treatment, were obtained for ex vivo studies from the A.O.U Città della Salute e della Scienza (Torino, Italy) and from the A.O.S. Croce e Carle (Cuneo, Italy) hospitals. The average age of patients was 57 years (range 25–86), with five males and 14 females. Supplementary Table S1 lists the characteristics of the patients included in this study. The data were collected in accordance with the Declaration of Helsinki and good clinical practice guidelines. The study was approved by the local Institutional Review Board (Comitato Etico Interaziendale A.O.U. Città della Salute e della Scienza di Torino—A.O. Ordine Mauriziano—A.S.L. TO1 and A.O.S. Croce e Carle, Cuneo, Italy). Full written informed consent was obtained from all patients involved in the study. Peripheral blood mononuclear cells and bone marrow mononuclear cells were separated by standard Ficoll-Hypaque density-gradient centrifugation. Mononuclear cells were cultured in DMEM medium supplemented with 20% fetal calf serum (FCS), 2 mM l-glutamine and 100-unit penicillin, 100 μg/ml streptomycin. According to the number of cells obtained from each sample, AML cells were used immediately for ex vivo experiments, or frozen in FCS with 20% DMSO.

### Cell lines

U937 and THP1 AML cell lines (from American Type Culture Collection, ATCC, Manassas, VA, USA) were grown in RPMI-1640 medium supplemented with 10% FCS; OCI-AML3 cells (kindly provided by G. Saglio, University of Torino, Italy) were cultured in DMEM medium supplemented with 20% FCS. Culture media were supplemented with 100-unit penicillin, 100 μg/ml streptomycin and 2 mM l-glutamine. Morphology of cell lines was monitored routinely, and mycoplasma contamination was excluded using a PCR-based assay. DNA fingerprinting analysis using 16 different, highly polymorphic short tandem repeat loci (PowerPlex 16 HS System, Promega) confirmed cell authenticity.

### Antibodies and reagents

The SY11B5 anti-CD157 (IgG1) mouse monoclonal antibody (mAb) was produced in-house and affinity-purified on protein G. RF3 anti-CD157 (IgG1) mAb produced in mouse was purchased from MBL (Voden-Medical, Meda, MB, Italy). The isotype control IgG1 produced in mouse (mIgG, clone MG1-45) was purchased from Biolegend (Aurogene, Roma, Italy). Purified murine IgG Fc fragments were from Jackson ImmunoResearch (West Grove, PA). Antibodies to phospho-mTOR Ser-2448 (D9C2, #5536), phospho-p70S6K Thr-389 (108D2, #9234), phospho-AKT Ser-473 (D9E, #4060), phosphor-GSK-3β Ser-9 (D85E12, #5558), phosho-ERK1/2 Thr-202/Tyr-204 (D13.14.4E, #4370), phosho-S6 ribosomal protein Ser-235/236 (91B2, #4857), phosho-4E-BP1 Thr-37/46 (236B4, #2855), mTOR (L27D4, #4517), AKT (40D4, #2920), GSX3β (27C10, #9315) PARP-1 (46D11, #9532), Caspase-3 (D3R64, #14220), Caspase-9 (C9, #9508), Bcl-XL (54H6, #2764), Bcl-2 (D55G8, #4223), Bim (C34C5, #2933), Bax (D2E11, #5023), Bak (D4E4, #12105), Mcl-1 (D35A5, #5453), HRP-conjugated anti-mouse IgG1 (#7076) and HRP-conjugated anti-Rabbit IgG1 (#7074) were obtained from Cell Signaling Technology (Euroclone, Milan, Italy). Antibodies to β-actin (MAB-24008-HRP) and β-tubulin (MAB-80142-HRP) were purchased from Immunological Sciences (Roma, Italy). AraC was purchased from Sigma-Aldrich, S63845 and ABT-199/venetoclax inhibitors from Selleck Chemicals (Aurogene, Roma, Italy). Both ABT-199 and S63845 were dissolved in DMSO. Fibronectin from human plasma, Fibrinogen and Collagen type I were from Sigma-Aldrich (Milano, Italy).

### Immunofluorescence staining and flow cytometry analysis

CD157 surface expression on AML samples and cell lines was assessed by flow cytometry (BD FACS Canto, BD Biosciences) by staining cells (3 × 10^5^/sample) with a PE-labeled mAb against CD157 (clone SY11B5, Invitrogen, Milan, Italy) for 20 min at 4 °C. Leukemic blasts were analyzed by multiparametric flow cytometry and gated based on CD45 expression (anti-CD45-APC/H7, BD Biosciences) and side scatter (CD45^dim^ and SSC^low^). To further characterize blast subpopulations the following antibodies were used: CD33-FITC, CD34-PE/Cy7, CD64-PE, CD117-PE, CD123-PE/Cy7, HLA-DR-FITC (Biolegend). Specifically, this back-gating allowed us to identify leukemic myeloid blasts (CD33^dim^, CD64−, CD117+, CD123+, HLA-DR+) and leukemic monocytic populations (CD33+, CD64++, CD117^low/neg^, CD123+, HLA-DR++). Data were analyzed using FlowJo software Version 10.6 (BD Biosciences). CD157 relative Mean Fluorescence Intensity (MFI) was normalized to the MFI of lymphocytes, which are CD157 negative. CD157 MFI ratio was calculated according to the formula: (MFI of CD157 in blasts − MFI of CD157 in lymphocytes)/MFI of CD157 in lymphocytes.

### CD157 gene knockdown by shRNA

CD157 expression in U937, THP1 and OCI-AML3 cell lines was silenced by lentiviral delivery of pLV-puro (Biosettia, San Diego, CA) encoding the BST-1 short-harpin RNA (shRNA) (target sequences: 5′-GAGTCAGACTGCTTGTATA-3′ (shCD157#1) and 5′-CCTGAGCGATGTTCTGTAT-3′ (shCD157#2) or a scrambled negative control (scrambled sequence: 5′-TTCTCCGAACGTGTCACGTT-3′), as previously described^24^.

### RNA extraction and RT-PCR

Total RNA was extracted from transduced AML cell lines using TRI reagent (Sigma-Aldrich), and quality and quantity checked with NanoDrop (ND-100, Thermo Fisher Scientific, Monza, Italy). 2 µg of RNA were reverse transcribed with the M-MLV Reverse Transcriptase (Invitrogen) and Oligo-dT primers (Sigma-Aldrich), as previously described^24^. PCR products were analyzed by electrophoresis on 1% agarose gel stained with Midori Green Advance DNA Stain (NIPPON Genetics Europe, Düren, Austria).

### Antibody-mediated CD157 stimulation

In selected experiments, primary AML cells (5 × 10^5^/ml) or cell lines (4 × 10^5^/ml) were treated for 24 h (unless otherwise indicated) at 37 °C in standard culture medium or in serum-free conditions, as indicated, with affinity purified SY11B5 anti-CD157 mAb (10 µg/ml), or with an isotype-matched mIgG (10 µg/ml), as control. Then, cells were washed once in RPMI 1640 medium and processed according to the indicated assay.

### Flow cytometry analysis of apoptosis

Apoptosis was determined by flow cytometry measurement of phosphatidylserine exposure using Annexin V-FITC. Briefly, AML cells were treated with the indicated stimulus or drug for 24 h, washed once in RPMI 1640 medium, and then incubated with 100 µl of Annexin V binding buffer containing 2 µl of Annexin V-FITC and 2 µl of Propidium Iodide (PI) (Kit #IK-90314, Immunological Sciences) for 20 min at room temperature in the dark. Annexin V fluorescence was determined with a BD FACS Canto (BD Biosciences) flow cytometer and the membrane integrity of cells was simultaneously assessed by PI exclusion method. Flow cytometry analysis was performed by FlowJo software Version 10.6 (BD Bioscience). At least three independent experiments were performed with AML cell lines. Patient samples were chosen on the basis of availability of adequate cell number and experiments were performed once.

### PrestoBlue viability assay

Primary AML cells (6 × 10^4^/well) or AML cell lines (5 × 10^4^/well) were seeded in 96-well plates in 100 µl of culture medium supplemented with FCS and treated as indicated. Drugs at the indicated dilutions were added to each well. At each time point, 10 µl/well of PrestoBlue reagent (Thermo Fisher Scientific) was added for 1 h at 37 °C, then fluorescence was measured by using the Infinite M200 Pro Microplate Reader (Tecan Italia, Cernusco sul Naviglio, MI, Italy) with excitation/emission wavelengths set at 560/590 nm. Cell viability was determined by comparing the fluorescence values before and after treatment and normalizing the data to controls. Half-maximal effective concentration (EC_50_) values were calculated by non-linear regression algorithms and a four-parameter logistic model with log-transformed data using GraphPad Prism 7 software.

### Western blot analysis and immunoprecipitation

Cells were washed and harvested in ice-cold phosphate-buffered saline and subsequently lysed in extraction buffer (50 mM Tris HCl pH7.4, 150 mM NaCl, 1% Triton-X 100, 0.5% Sodium Deoxycholate, 1 mM EDTA, 1 mM EGTA, 10 mM NaF, 0.1% SDS) supplemented with 1 mM Na_3_VO_4_ and protease inhibitor cocktail. Protein concentration was determined using the Bradford assay (BioRad Laboratories, Segrate, MI, Italy). Equal amounts of proteins were separated by an Any-kD Critereon TGX Stain-free PreCast gel (BioRad) and transferred to nitrocellulose membranes (Trans-blot Turbo Transfer pack, BioRad). After blocking with 2% BSA for 1 h, membranes were cut to minimize antibody use and each strip was separately probed with the indicated primary antibody, followed by the appropriate HRP-conjugated secondary antibody. Immunoreactive proteins were detected using enhanced chemiluminescence, images were captured with a ChemiDoc XRS + System. Band densitometry measurements were performed with Image Lab Software (BioRad).

For immunoprecipitation, after the indicated treatments, cell extracts were obtained using lysis buffer (containing 50 mM Tris HCl pH7.4, 150 mM NaCl, 1% NP-40, 0.5% Sodium Deoxycholate) supplemented with 5 mM EDTA, 10 mM NaF, 1 mM Na_3_VO_4_, 1 mM DTT and protease inhibitor cocktail. An equivalent of 1 mg/sample of protein extracts was incubated overnight at 4 °C with anti-Mcl-1 (D2W9E, #94296) or anti-Bim (C34C5, #2933) antibodies (Cell Signaling, Euroclone, Milan, Italy), and subjected to immunoprecipitation with 20 µl of anti-rabbit IgG-coated Sepharose beads (Cell Signaling) for 3 h at 4 °C. Proteins were eluted by boiling samples for 5 min at 95 °C with 3× SDS reducing sample buffer and then analyzed by Western blotting. Equal amounts (20 µg) of lysate was prepared as described above, and used as whole-cell lysate control.

### Statistical analysis

Unless otherwise indicated, results of in vitro experiments are expressed as means ± SEM of at least three independent experiments. Comparisons among multiple groups were performed with both one-way and two-way ANOVA models. Differences between two groups were analyzed using unpaired two-tailed Student’s *t*-test. When the data were not normally distributed, the comparison of variables was performed with a Wilcoxon signed rank test for paired data or with a Mann–Whitney test for unpaired data. All statistical analyses were performed using GraphPad Prism 7.0 (GraphPad Software, San Diego, CA). For all analyses, *p* values < 0.05 were considered statistically significant (*p < 0.05, **p < 0.01, ***p < 0.001, ****p < 0.0001 versus control, and ns for not significant).

## Results

### CD157 promotes primary AML cell survival ex vivo

Primary AML cells maintained ex vivo in standard culture conditions spontaneously undergo apoptosis, caused by absence of soluble mediators and lack of interactions between leukemic cells and both cellular and extracellular stromal components^27,28^. To explore the potential role of CD157 signaling in AML cell survival in vitro, we used BM and peripheral blood samples from 19 newly-diagnosed AML patients (Supplementary Table S1) showing variable levels of CD157 expression (Supplementary Fig. S1).

**Figure 1:**
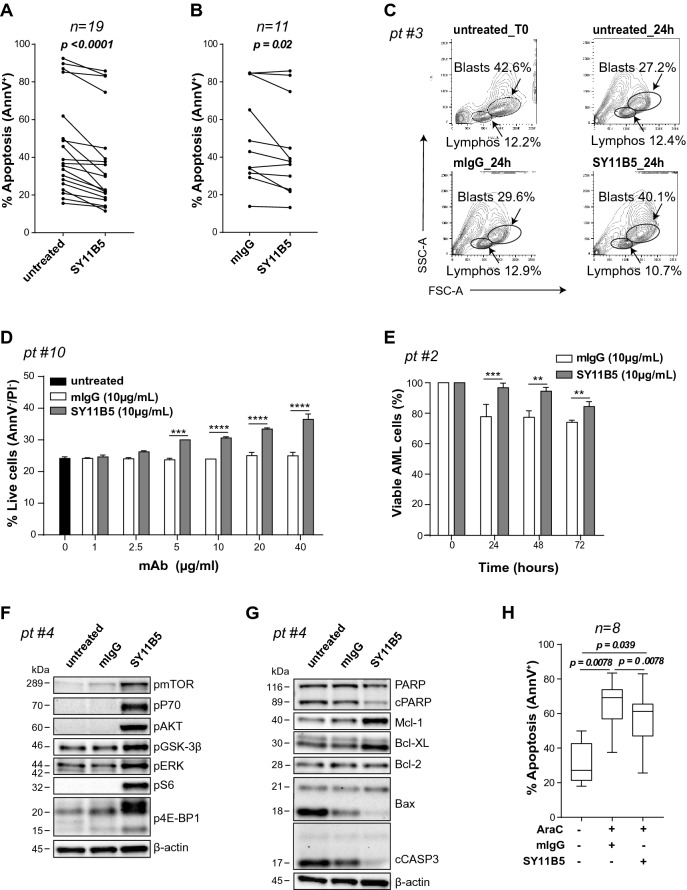
CD157 modulates viability in primary AML cells. Mononuclear cells from AML patients were maintained ex vivo for 24 h under standard culture conditions (untreated) or treated with (**A**, **B**) SY11B5 anti-CD157 mAb or (**B**) control mIgG, then were subjected to AnnexinV/PI staining and flow cytometry analysis. Each dot represents a single patient. *p* < 0.0001 and *p* = 0.02, Wilcoxon’s signed-rank test. (**C**) Flow cytometry analysis of a bone marrow sample from a representative AML patient. Density plots show the distribution of each population composing the mononuclear cell fraction (X axis = FSC, Y axis = SSC) at time zero (T0), after 24 h in standard medium (untreated, top panels), with mIgG (bottom left panel) or SY11B5 anti-CD157 mAb (bottom right panel). Lymphocytes (FSC^low^/SSC^low^) and blasts (FSC^dim^/SSC^dim^) were gated according to morphological parameters. Percentage indicates the frequency of each population compared to total acquired events. (**D**) Pro-survival effect of increasing concentrations of SY11B5 anti-CD157 mAb or mIgG at 24 h in primary AML cells from a representative patient. Black bar represents the percentage of viable untreated cells. (**E**) Pro-survival effect of SY11B5 anti-CD157 mAb compared with mIgG. The percentage of live cells at each time point compared to time zero is shown. The graph is a representative PrestoBlue assay performed in quadruplicate. **p < 0.01, ***p < 0.001, ****p < 0.0001, two-way ANOVA with Sidak’s multiple comparison test. Western blot analysis of phosphorylation levels of the indicated proteins of (**F**) PI3K/AKT/mTOR pathway and (**G**) Bcl-2 apoptotic pathway, following CD157 stimulation for 24 h with SY11B5 anti-CD157 (10 µg/ml) mAb or mIgG (10 µg/ml), used as isotype-matched control. β-Actin was used as loading control. A representative Western blot is shown out of four patients analysed with similar results. Samples analysed in panel F and G derive from the same experiment and were processed in parallel. Uncropped blot image is provided in Supplementary Fig. S8. (**H**) AML samples were treated for 24 h with vehicle or AraC (10 µM) in the presence of anti-CD157 mAb or mIgG (both at 10 µg/ml). The percentage of apoptotic cells was measured by AnnexinV/PI staining and flow cytometry analysis. *p* values were determined by Wilcoxon’s signed-rank test.

To test for CD157-driven effects on AML cell viability, cells were maintained for 24 h in DMEM culture medium with 20% FCS in the presence of SY11B5 agonistic anti-CD157 mAb, or without (untreated control). Then, cell apoptosis was quantified by Annexin V/PI double staining and flow cytometry analysis. After 24 h of ex vivo culture, AML cell apoptosis ranged from 15.6 to 92.5%. However, for every sample tested, the percentage of apoptotic cells (e.g., Annexin V+) decreased after CD157 mAb treatment with respect to untreated controls (Fig. 1A). In contrast, treatment with an isotype-matched control mAb (mIgG) had no protective effect against apoptosis (Fig. 1B and Supplementary Fig. S2A). Five AML cell samples showing > 50% apoptosis ex vivo had low expression of CD157; however, the correlation between CD157 expression and ex vivo survival was not statistically significant, at least in the small number of samples analysed (Supplementary Fig. S2B).

To determine whether the pro-survival effect mediated by CD157 was specific to leukemic blasts, flow cytometry analysis was carried out on cell subsets identified through forward scatter (FSC) and side scatter (SSC) parameters at baseline (T0) and following 24 h ex vivo culture in the presence or absence of anti-CD157 mAb (or control mIgG) (Fig. 1C). After 24 h, the percentage of viable blasts remained almost unchanged (42.6% vs 40.1%) in the CD157 antibody-treated sample. However, in untreated and mIgG-treated samples, the percentage of viable blasts was notably reduced (42.6% vs 27.2% and 29.6%, respectively). In addition, we observed that CD157-mediated survival was dose-dependent (Fig. 1D) and extended in time to at least 72 h (Fig. 1E). These results indicate that CD157 contributed to leukemic blast survival.

### CD157 regulates intracellular signal transduction and apoptosis

To decipher the molecular mechanisms through which CD157 promoted AML blast survival, we analyzed the intracellular signals elicited by CD157 mAb targeting in AML cells. We focused on PI3K/AKT/mTOR and mitogen-activated protein kinase (MAPK)/extracellular signal kinase (ERK) signal transduction pathways, known to be activated through CD157 targeting in monocytes^20^ and also frequently deregulated in AML^29,30^.

Antibody targeting of CD157 expressed in primary leukemic cells for 24 h induced phosphorylation, as visualized by Western blot analysis, of: (1) mTOR and its downstream substrates p70S6K, pS6 ribosomal protein and 4E-BP1; (2) ERK and (3) AKT at its Ser-473, leading to (4) Ser-9 inactivating phosphorylation of Glycogen Synthase Kinase 3β (GSK-3β) (Fig. 1F). GSK-3β is a major AKT target, implicated in several cellular processes, including the regulation of cell death^31^, and GSK-3β was previously found to be associated with poor survival outcome in AML patients^32^. Next, to gather evidence that CD157 stimulation could modulate apoptosis, AML cells were treated with anti-CD157 mAb or mIgG as before, and analysed by Western blot for expression of proteins belonging to the Bcl-2-family. Although Bcl-2 expression was marginally affected, the anti-apoptotic proteins Mcl-1 and Bcl-XL were strongly upregulated while the pro-apoptotic protein Bax was clearly downregulated following CD157 antibody-binding. Moreover, CD157 stimulation reduced the proteolytic cleavage of Caspase-3 and its substrate PARP-1, considered to be hallmarks of apoptosis (Fig. 1G). Collectively, these results highlighted that by activating the PI3K/AKT/mTOR and MAPK signaling pathways, CD157-mediated intracellular signals can contrast spontaneous apoptosis in primary AML cells ex vivo and promote cell survival.

### CD157 modulates AraC-mediated apoptosis in primary AML blasts

Next, we investigated if CD157 signaling could protect AML blasts from apoptosis induced by therapeutic drugs, such as AraC, a mainstay of AML treatment, thus potentially interfering with the efficacy of chemotherapy. To address this issue, eight bone marrow samples were treated ex vivo with anti-CD157 mAb or with control mIgG immediately before addition of 10 µM AraC. Cell apoptosis was determined after 24 h. Ligation of CD157 by SY11B5 mAb significantly reduced the number of apoptotic cells in the AML samples, compared to the corresponding controls treated with mIgG (Fig. 1H), indicating that CD157 signaling can interfere with AraC-induced apoptosis in primary AML cells.

### CD157 prevents apoptosis induced by nutrient deprivation in AML cell lines

To combine the antibody-mediated approach with genetic manipulation of CD157 expression in AML cells, we extended our studies using U937, THP1 (both AML FAB M5 subtype) and OCI-AML3 (AML FAB M4 subtype) human AML cell lines which express variable levels of CD157 mRNA and protein (Supplementary Fig. S3A,B,D) almost entirely consisting of the canonical CD157/BST-1 protein from the 9-exon transcript encoded by *BST1*^33^ (Supplementary Fig. S3C).

**Figure 2:**
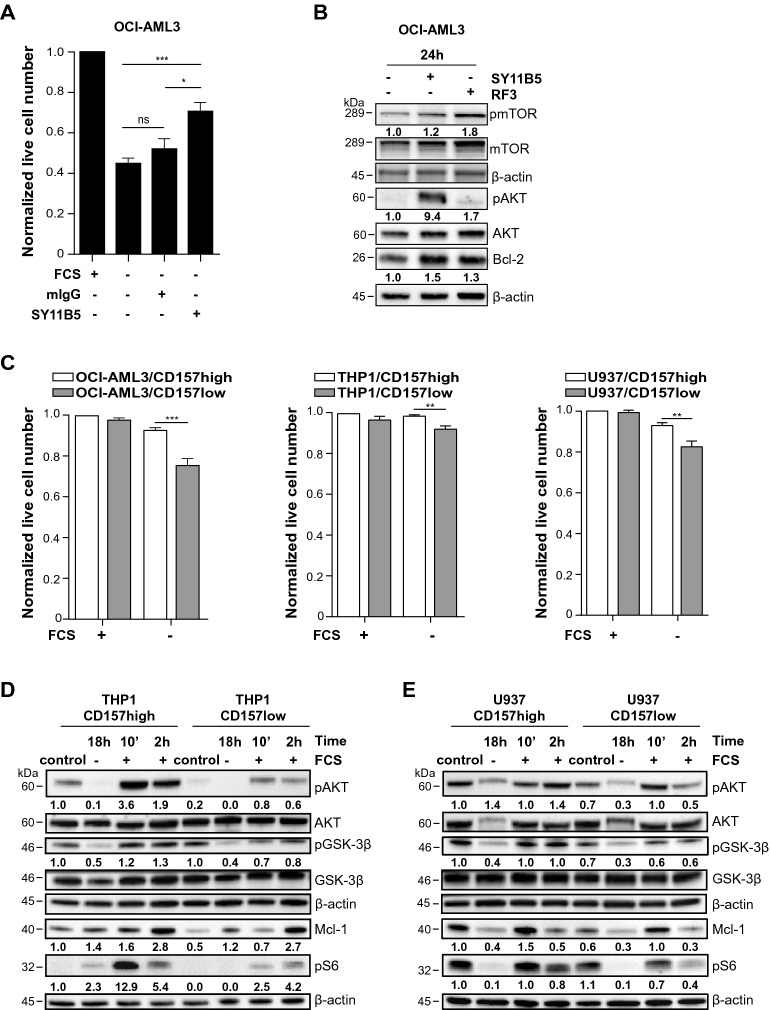
CD157 modulates cellular stress responses in AML cell lines. (**A**) OCI-AML3 cells were cultured overnight in the absence of FCS, then the SY11B5 anti-CD157 mAb or mIgG (both at 10 µg/ml) were added for 24 h. Cell viability was measured by AnnexinV/PI staining and flow cytometry analysis. Histograms show the effect of nutrient deprivation on cell viability expressed as fold change of viable cells compared to cells maintained in standard culture conditions, and are the mean ± SEM of six independent experiments performed in quadruplicate. *p < 0.05, ***p < 0.001, *ns* = not significant, one-way ANOVA with Tukey’s multiple comparison test. (**B**) OCI-AML3 were cultured overnight in serum-free medium then, SY11B5 or RF3 mAbs to CD157 (10 µg/ml) were added for 24 h. Whole cell lysates were subjected to Western blotting and probed with the indicated antibodies. Blots were re-probed using antibodies against the total proteins. β-Actin was used as loading control. Numbers below blots indicate fold change in the expression of each protein relative to untreated control, normalized to the corresponding β-actin and total protein (mTOR and AKT). Uncropped images are provided in Supplementary Fig. S9 (**C**) CD157-high or low OCI-AML3, THP1 and U937 cells were cultured in standard conditions or in serum-free medium for 24 h, and then subjected to AnnexinV/PI staining and flow cytometry analysis. Results are expressed as fold change of viable cells normalized to CD157-high cells maintained in standard culture conditions and are the mean ± SEM of three experiments. *p < 0.05, **p < 0.01, one-way ANOVA with Tukey’s multiple comparison test. (**D**) CD157-high and CD157-low THP1 cells and (**E**) CD157-high and CD157-low U937 cells were maintained for 18 h in FCS-free culture medium, then were stimulated with FCS for 10 min or 2 h. Whole cell lysates were subjected to Western blotting and probed with the indicated antibodies. Blots were re-probed using antibodies against the total proteins. β-Actin was used as loading control. Numbers below blots indicate fold change in the expression of each protein relative to untreated CD157-high cells, normalized to the corresponding β-actin and total protein (AKT and GSK3β). Panels (**D**) and (**E**) show one Western blot representative of three independent experiments performed. Uncropped blot images are provided in Supplementary Fig. S9.

To evaluate their propensity for apoptosis under stress and the capacity of CD157 to rescue, the AML cell lines were placed in culture medium without FCS, a strategy known to induce activation of the mitochondrial apoptotic pathway in leukemia cells^34^. After 24 h in serum-free medium, cell viability was measured and found to be significantly reduced in OCI-AML3 cells (Fig. 2A), whereas, U937 and THP1 cells were only marginally affected (Supplementary Fig. S4A). However, treatment with anti-CD157 mAb significantly increased OCI-AML3 survival (Fig. 2A). Similar results were obtained by pretreating OCI-AML3 cells with saturating concentrations of murine IgG Fc fragments, thus excluding the potential interference of Fc receptor-mediated effects (Supplementary Fig. S4B). By Western blot, CD157-stimulated OCI-AML3 cells showed increased phosphorylation of mTOR and AKT, and increased expression of Bcl-2, compared to the untreated control. Similar results were obtained with RF3 anti-CD157 mAb (Fig. 2B), which is known to bind to a different epitope.

To pinpoint the emerging role of CD157 in AML, we generated stable CD157 knockdown versions from the parental U937, THP1 and OCI-AML3 cell lines using lentiviral vectors expressing two shRNA (shCD157#1 and shCD157#2) targeting CD157 or a scramble control sequence that conserved endogenous expression of CD157 (CD157high cells). CD157 expression in the genetically engineered cell lines was assessed by RT-PCR (Supplementary Fig. S3A,B) and flow cytometry (Supplementary Fig. S3D). We selected the cell lines with lowest CD157 expression (CD157-low) for further analysis to compare CD157high versus CD157low AML cells. Under standard culture conditions, the CD157-high and the CD157-low version of the AML cell lines showed similar proliferation ability (Supplementary Fig. S3E). However, when cultured in the absence of FCS for 24 h, the CD157-low cells showed significantly fewer live cells than the CD157-high cells, indicating that CD157 raises the apoptotic threshold in these leukemic cells (Fig. 2C).

We previously demonstrated that CD157-fibronectin interaction activates the PI3K/AKT/mTOR signaling pathway^17,18^. To evaluate activation of this pathway, we selected the CD157-high and CD157-low version of THP1 and U937 cells (OCI-AML3 cells was excluded as the parental line has lower CD157 expression). After overnight starvation, cells were maintained in one of the following conditions: (1) standard culture, (2) without FCS, (3) with 10% FCS for 10 min and (4) with 10% FCS for 2 h. Cell extracts were then analysed by Western blot. In standard culture conditions, both CD157-high THP1 and U937 expressed higher Mcl-1 protein levels compared to their respective CD157-low counterparts. In the presence of FCS, CD157-high THP1 and U937 showed more robust phosphorylation of AKT at Ser-493, GSK-3β kinase at Ser-9 and pS6 ribosomal protein at Ser-235/236 and increased the expression of Mcl-1 (especially after 10 min of exposure to FCS) with respect to their CD157-low versions (Fig. 2D,E). The same experiment was repeated in CD157-high and CD157-low THP1 cells following treatment with purified fibronectin, fibrinogen or collagen type 1 that bind CD157, with similar results. Indeed, exposure to purified ECM proteins induced higher phosphorylation of AKT, GSK-3β kinase and pS6 ribosomal protein (especially after 10 min) and increased expression of Mcl-1 (after 2 h) in CD157-high than in CD157-low cells (Supplementary Fig. S5). Taken together, these results showed that stimulation of CD157 by mAb binding or FCS/ECM protein stimulation significantly activated the PI3K/AKT/mTOR pathway while inactivating GSK-3β kinase, leading to upregulation of Mcl-1.

### CD157 knockdown enhances AML cells sensitivity to AraC-induced apoptosis

**Figure 3:**
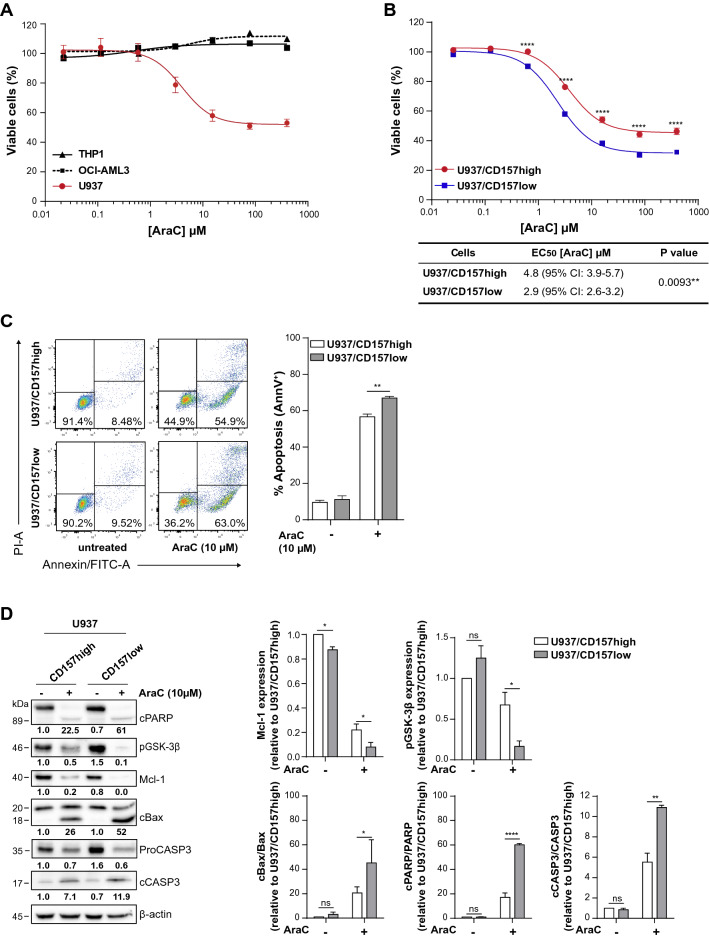
CD157 knockdown increases AraC-induced cell death in U937 cells. (**A**) THP1 (black line), OCI-AML3 (dashed black line) or U937 (red line) cells were treated with increasing concentrations of AraC for 24 h to assess the sensitivity of each AML cell line to AraC. (**B**) The sensitivity of CD157-high (red line) versus CD157-low (blue line) U937 cells to AraC was compared by Presto Blue assays. Six replicates for each condition have been performed. Results are the mean ± SEM of four experiments. ****p < 0.0001, Student’s t-test. The EC_50_ for U937 cells with high or low CD157 was calculated using the GraphPad software (see “Materials and methods”). (**C**) Representative dot plot (left) and histogram (right) of CD157-high and CD157-low U937 cells treated with AraC for 24 h, obtained by flow cytometry analysis after AnnexinV/PI staining. Histograms show the mean ± SEM of eight independent experiments. **p < 0.01, two-way ANOVA with Sidak’s multiple comparison test. (**D**) U937/CD157-high and U937/CD157-low were treated with AraC for 24 h, then whole cell lysate was subjected to Western blotting. Numbers below blots indicate fold change in the expression of each protein relative to untreated CD157-high cells, normalized to the corresponding β-actin and full length protein (PARP, Bax and Caspase-3). β-Actin was used as loading control. Left panels: the levels of Mcl-1, pGSK-3β, cBax, cPARP, and cCaspase-3 were determined by densitometry analysis and are the mean of three experiments run in parallel. Uncropped blot is provided in Supplementary Fig. S10. Data are expressed as the mean ± SEM of band densities of western blots from three separate experiments. *p < 0.05, **p < 0.01, ****p < 0.0001, *ns* = not significant, two-way ANOVA with Sidak’s multiple comparison test.

Having established that CD157 rescues AML cell from apoptosis, we then analysed the impact of CD157 signaling in the response of AML cell lines to chemotherapy. First, cell viability was determined in THP1, U937 and OCI-AML3 cells exposed to increasing concentrations of AraC for 24 h. In the experimental conditions adopted, U937 cells were sensitive to AraC treatment, while THP1 and OCI-AML3 cells were resistant (Fig. 3A). Hence, subsequent experiments were performed in U937 cells, further showing that U937/CD157-low were significantly more sensitive to AraC toxicity than U937/CD157-high cells (Fig. 3B). The half-maximal effective concentration (EC_50_) of AraC decreased from 4.8 µM in U937/CD157-high cells to 2.9 µM in U937/CD157-low cells (*p* = 0.0093). Moreover, following treatment with 10 µM AraC, the percentage of apoptotic U937/CD157-low cells was significantly higher than that of U937/CD157-high cells (apoptotic cells = 66.0% ± 2.9 in CD157-low cells versus 55.9% ± 3.1 in CD157-high cells; *p* < 0.01, n = 8) (Fig. 3C). Overall, these findings corroborate the results inferred from the experiments performed in patient-derived primary AML cells, and confirmed that CD157 signaling decreases the sensitivity of AML cells to AraC toxicity.

### CD157 reduces the sensitivity of AML cells to AraC through the modulation of the intrinsic apoptotic pathway

Inhibition of apoptosis plays a central role in modulating malignant cell responses to anti-cancer therapy. Therefore, we investigated if CD157 signaling could decrease the sensitivity of U937 cells to AraC by modulating apoptosis. Treatment of U937 cells with AraC is known to induce apoptosis by Mcl-1 degradation and Bax cleavage with generation of a pro-apoptotic fragment of 18-kDa^35^. By Western blot, U937/CD157-high cells showed higher constitutive levels of Mcl-1 than U937/CD157-low cells, which was only partly degraded after 24-h AraC treatment (Fig. 3D). Moreover, U937/CD157-high cells retained some degree of GSK-3β phosphorylation at Ser-9, which contributes to Mcl-1 stabilization^36^. Corroborating the reduced apoptosis observed in U937/CD157-high cells exposed to AraC, we found reduced activation of Bax pro-apoptotic effector and reduced cleavage of Caspase‐3 and PARP-1, compared to U937/CD157-low cells (Fig. 3D). These findings confirmed that signaling through CD157 modulates AML cell sensitivity to AraC by inhibiting apoptosis.

**Figure 4:**
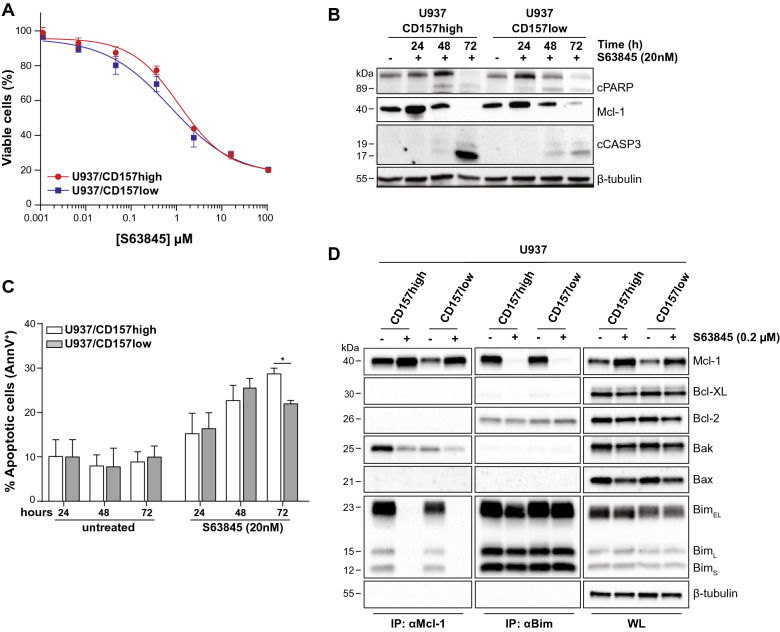
CD157 promotes survival of leukemic blasts through Mcl-1 upregulation. (**A**) CD157-high (red line) or CD157-low (blue line) U937 cells were treated for 24 h with increasing concentrations of S63845, then cell viability was determined by Presto Blue assays. Data represents the percentage of live cells compared to control cells treated with vehicle and are the mean ± SEM of three experiments performed in six replicates. (**B**) Western blot analysis of PARP-1, Mcl-1 and cleaved Caspase-3 in CD157-high or CD157-low U937 cells treated with S63845 for 24, 48 or 72 h. β-Tubulin was used as loading control. Uncropped blot images are provided in Supplementary Fig. S11. (**C**) CD157-high or CD157-low U937 cells were treated for up to 72 h with S63845. Cell apoptosis was measured by flow cytometry analysis after AnnexinV/PI staining. Results are the mean ± SEM of three experiments. *p < 0.05, two-way ANOVA with Sidak’s multiple comparison test. (**D**) U937/CD157-high or U937/CD157-low cells were treated with S63845 for 24 h, then lysed and subjected to immunoprecipitation with anti-Mcl-1 or anti-Bim antibody, respectively. The interactions with the indicated proteins were analyzed by immunoblotting. β-Tubulin was used as loading control. One representative Western blot is shown (n = 3). The whole cell lysates (WL) show the protein expression levels. Uncropped blot images are provided in Supplementary Fig. S11.

To strengthen these observations, we assessed apoptosis in U937 treated with either ABT-199/venetoclax, a Bcl-2-specific inhibitor, or S63845, which specifically inhibits Mcl-1. Consistent with previous observations^37^, U937 cells were resistant to ABT-199 (as were THP1 and OCI-AML3 cells^6,38^, data not shown), regardless of CD157 expression levels (Supplementary Fig. S6A). However, combined ABT-199 and AraC treatment significantly increased apoptosis induced by AraC used as single drug, both in U937 with high or low CD157 (Supplementary Fig. S6B). U937 cells were sensitive to S63845, although we found no correlation between sensitivity and Mcl-1 expression, as also observed in previous studies^39,40^ nor CD157 expression (Fig. 4A). To assess the relationship between CD157-mediated anti-apoptotic effects and Mcl-1, U937 cells were treated with 20 nM S63845 for 24, 48 or 72 h and then analyzed by Western blot. After 24 h, Mcl-1 protein levels increased in both U937/CD157-high and U937/CD157-low cells (Fig. 4B), as S63845 is known to prolong Mcl-1 half-life^39–41^. However, accumulation of Mcl-1 was greater in CD157-high than in CD157-low cells. After 48–72 h, Mcl-1 was progressively degraded, while Caspase-3 and PARP-1 were cleaved, indicating activation of apoptosis. Cleavage of Caspase-3 was evident in U937/CD157-high but less so in U937/CD157-low cells. Consistently, after 72-h exposure to S63845, the percentage of cells undergoing apoptosis was significantly higher in CD157-high compared to CD157-low U937 cells (Fig. 4C).

The anti-apoptotic Bcl-2 family proteins act by binding and inhibiting pro-apoptotic proteins, sensors of cellular stress (the BH3-only proteins) and effectors of apoptosis (Bax and Bak)^42^. To unravel the anti-apoptotic effect of CD157 expression in AML cells, using U937 cells with high or low CD157 we immunoprecipitated Mcl-1 and found it was associated with Bim (Fig. 4D). In CD157-high cells, Mcl-1 also sequestered Bak. In contrast, the Mcl-1/Bak interaction was almost negligible in CD157-low cells. Treatment with 200 nM S63845 displaced Bim from Mcl-1 and drastically destabilized Mcl-1/Bak association. Reciprocal immunoprecipitation using a Bim antibody confirmed that in U937 cells Bim was mainly associated with Mcl-1, while it was only minimally associated with Bcl-2, regardless of the expression of CD157.

These data indicate that CD157 promotes survival of leukemic blasts through Mcl-1 upregulation, and suggest that sequestration of Bak by Mcl-1 might represent the primary CD157-mediated anti-apoptotic mechanism in AML cells.

### Mcl-1 inhibition improves therapeutic efficacy of AraC in CD157-high AML cells

**Figure 5:**
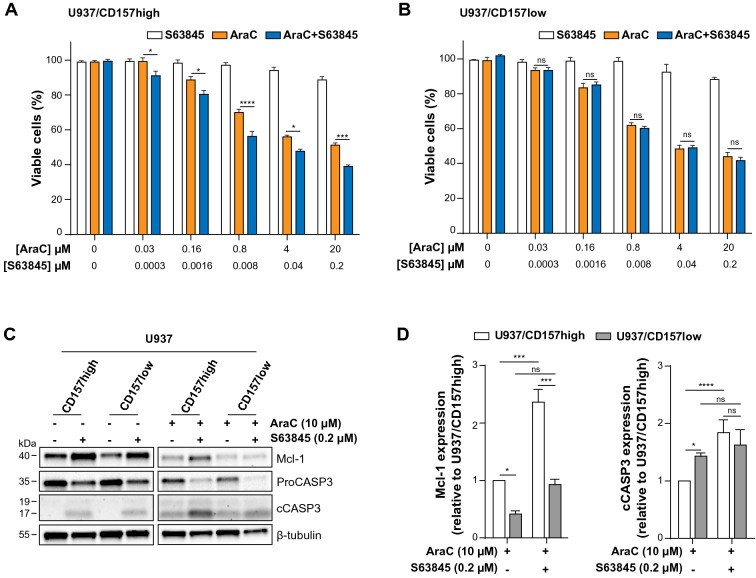
Mcl-1 inhibition improves therapeutic efficacy of AraC in CD157-high AML cells. (**A**) U937**/**CD157-high or (**B**) U937/CD157-low cells were treated for 24 h with increasing concentrations of S63845 or AraC as single drugs or in combination, at constant ratio S63845:AraC = 1:100. Cell viability was measured by PrestoBlue assays and was expressed as fold change of viable cells compared to cells maintained in standard culture conditions. (**C**) Western blot analysis of U937/CD157-high or U937/CD157-low cells left untreated or treated for 24 h with S63845 or AraC as single agents or in combination. Uncropped blot images are provided in Supplementary Fig. S12. (**D**) The levels of Mcl-1 (left), and cleaved Caspase-3 (right) proteins determined by densitometry analysis was compared in CD157-high (white bars) or CD157-low (grey bars) U937 cells treated with AraC as single drug or in combination with S63845 for 24 h. Relative band intensities of the target proteins were measured by densitometric analysis. Results are means ± SEM of band densities of western blots from three separate experiments and are expressed as fold change over AraC-treated U937/CD157-high cells. *p < 0.05, ***p < 0.001, ****p < 0.0001, *ns* = not significant, two-way ANOVA with Sidak’s multiple comparison test.

Overexpression of Mcl-1 has been implicated in resistance to both chemotherapy and to BH3-mimetics targeting Bcl-2 and Bcl-XL^43^, arousing considerable interest toward Mcl-1 as a therapeutic target in AML. Since Mcl-1 inhibitors are most likely to be effective in AML therapy when used in combination with a “priming” agent^44^, we compared the efficacy of S63845 in combination with AraC in U937 cells with high or low CD157. Cells were treated for 24 h with increasing concentrations of AraC either as single agent or in combination with increasing concentrations of S63845, maintaining a constant ratio between the two drugs (S63845:AraC = 1:100). Cell viability was then determined by PrestoBlue assays, which measures metabolic activity. The results showed that in CD157-high, but not in CD157-low U937 cells, the addition of S63845 significantly enhanced AraC-induced cell death at all doses considered, with respect to AraC alone (Fig. 5A,B). Western blot analysis showed that S63845 enhanced the activation of Caspase-3 induced by AraC, especially in U937/CD157-high cells (Fig. 5C). Densitometric measurements of the amount of Mcl-1 and cleaved Caspase-3 confirmed that AraC-treated U937/CD157-high cells had higher levels of Mcl-1 (Fig. 5D, left) and lower levels of cleaved Caspase-3 (Fig. 5D, right) compared to CD157-low cells. This amply reflects the reduced sensitivity to AraC of U937/CD157-high cells. S63845 was able to significantly increase AraC-induced Caspase-3 cleavage in CD157-high but not in CD157-low U937 cells (Fig. 5D). Indeed, following combined treatment with S63845 and AraC for 24 h, the different sensitivity to AraC of U937/CD157-high compared to CD157-low cells was abrogated (Supplementary Fig. S7).

## Discussion

In this study, we identified the biological function of CD157 in AML showing that CD157 favors survival and confers protection from stress or drug-induced apoptosis in primary AML blasts and AML cell lines. Two different experimental approaches support this notion. Firstly, antibody-directed binding of CD157 supported survival and reduced AraC-induced apoptosis in primary AML blasts ex vivo. Secondly, knockdown of CD157 increased apoptosis in AML cell lines exposed to nutrient deprivation conditions, and increased their sensitivity to AraC toxicity. These findings are in line with previous studies from our laboratory demonstrating that high levels of CD157 expression can promote tumor cell survival and induce drug resistance in non-hematological tumors, namely epithelial ovarian cancer^22,45^ and malignant pleural mesothelioma^23^, eventually strengthening tumor aggressiveness. Further evidence derives from the study by Mirkowska et al.^46^ of the B-cell precursor acute lymphoblastic leukemia (BCP-ALL) surface proteome in which CD157 was identified as one of a restricted set of markers with significantly higher expression in BCP-ALL cells with respect to normal bone marrow B cell populations.

The correlation between high CD157 expression levels and the group of patients with “adverse prognosis” according to the European Leukemia Net (ELN) classification-2017^47^ was reported in a small cohort of patients with AML^25^. Although is a preliminary observation, this report supports the idea that CD157 expression is associated with aggressive disease progression in vivo.

**Figure 6:**
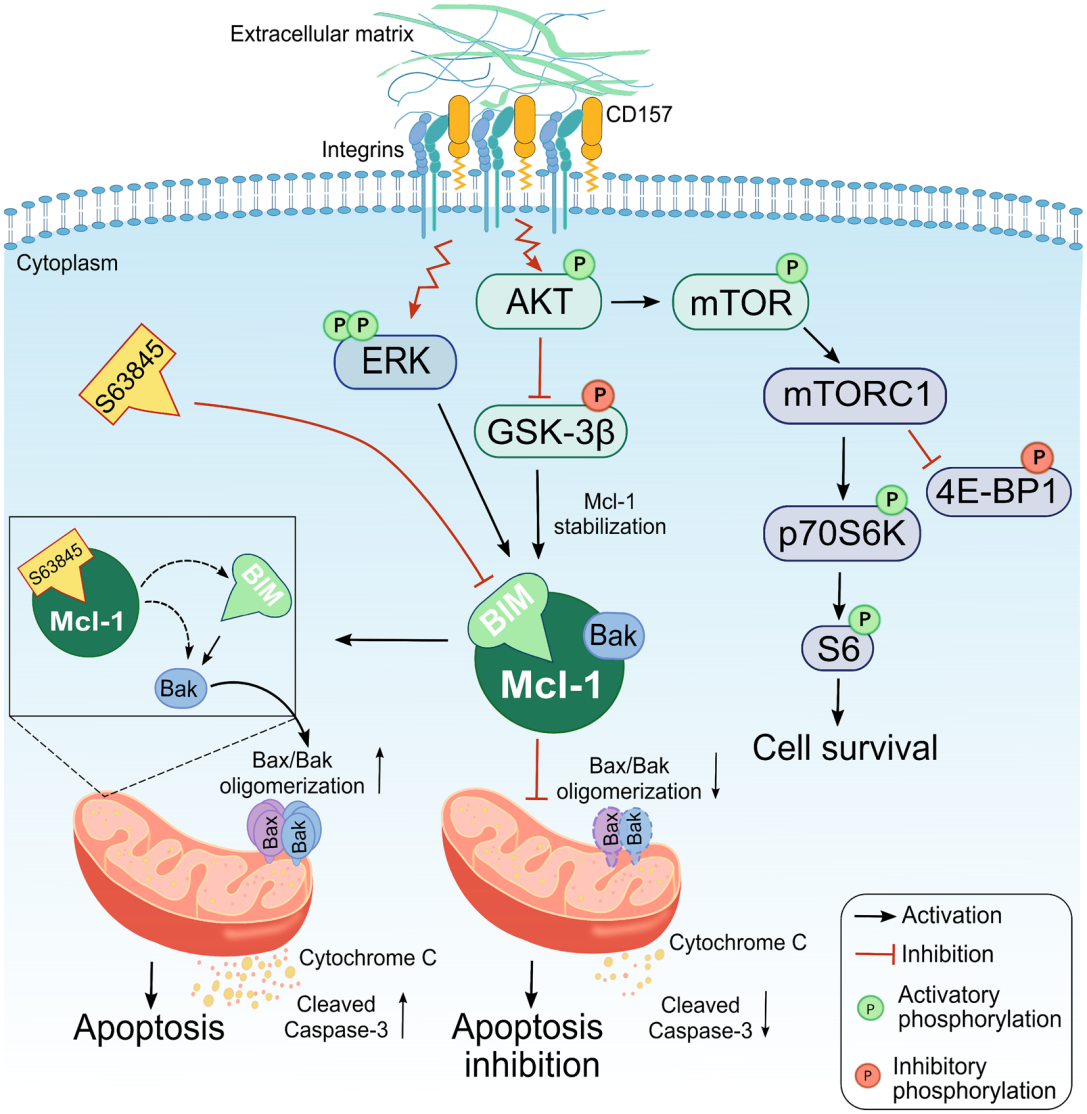
Schematic representation of CD157-mediated survival in AML cells. CD157 binding to fibronectin in AML cells promotes structural and functional interaction of CD157 with specific integrins. This activates intracellular signals leading to phosphorylation of AKT, mTOR and its downstream substrates p70S6K, pS6 ribosomal protein, 4E-BP1, and ERK. In turn, phosphorylated AKT inactivates its major target GSK-3β, which loses its pro-apoptotic function and favors the stabilization of Mcl-1. The increased expression of Mcl-1 strengthens its ability to sequester Bim and Bak, thus precluding the Bak/Bax oligomerization at the mitochondrial outer membrane, which is required for cytochrome c release and is considered a key step in apoptosis. S63845 binds to the BH3-binding groove of Mcl-1 releasing Bim and Bak and enabling the activation of the Bax/Bak-dependent apoptotic pathway. This results in outer mitochondrial membrane permeabilization and release of cytochrome c into the cytosol leading to activation of the caspase cascade.

In agreement with previous studies clearly delineating the ability of CD157 to act as a signaling molecule^20,21,33^, our data showed that CD157 exerts its pro-survival effects in AML cells by transducing signals from external cues. Indeed, in primary AML cells, CD157 stimulation by antibody binding activated the PI3K/AKT/mTOR and MAPK/ERK pathways, while inactivating GSK3-β kinase thus leading to extended ex vivo survival and reduced sensitivity to AraC treatment. In addition, stronger phosphorylation of AKT, GSK-3β, mTOR and its downstream effectors was found in CD157-high versus CD157-low AML cell lines exposed to FCS (as source of ECM proteins) or to purified ECM proteins that bind CD157^17^. PI3K/AKT/mTOR and MAPK/ERK intracellular signaling pathways, as well as GSK3-β, regulate the expression and activity of apoptotic proteins ultimately ruling the balance between survival versus death of cells^48^. In leukemic cells, aberrant activation of these intracellular signals converge towards apoptosis escape, favoring tumor growth and eventually leading to resistance to chemotherapy^49^. The results of this study unveiled that CD157 takes part to the cross-talk between the PI3K/AKT/mTOR pathway and apoptosis, contributing to leukemic cell survival. In particular, CD157-mediated intracellular signals enhanced both Mcl-1 and Bcl-XL anti-apoptotic proteins; in contrast, they decreased Bax pro-apoptotic protein and prevented Caspase-3 activation, thus mitigating the apoptotic response in primary AML cells. Accordingly, knockdown of CD157 in U937 and THP1 cells reduced Mcl-1 and resulted in increased sensitivity to AraC toxicity in U937 cells. In U937 cells, the pro-apoptotic protein Bim was primarily bound to Mcl-1, while binding of Bim to Bcl-2 was barely detectable. In addition, in CD157-high U937 cells, Mcl-1 sequestered the pro-apoptotic effector protein Bak precluding the activation of the Bax/Bak-dependent mitochondrial apoptosis^50^. Consistently, treatment with S63845, which binds with high affinity to the BH3-binding groove of Mcl-1^39,51^, displaced Bim and Bak bound to Mcl-1 resulting in activation of the Bax/Bak-mediated apoptosis^52^ (Fig. 6). Used in combination with AraC, very low doses of S63845 were sufficient to significantly increase the therapeutic efficacy of AraC in CD157-high but not in CD157-low AML cells. Overall, these findings demonstrated that CD157-driven intracellular signals upregulates Mcl-1, tilting the balance of leukemic cell homeostasis toward apoptosis evasion and raising their sensitivity threshold to AraC treatment, in vitro.

Remarkable clinical success with the Bcl-2 inhibitor venetoclax has revolutionized the treatment of hematological malignancies, including AML^6,53^. Several landmark trials have demonstrated that venetoclax is effective in treating AML when combined with conventional cytotoxic agents^54^. However, a significant minority of patients are refractory. In addition, the majority of patients that do achieve remission, eventually become resistant and relapse^55^. The upregulation of Mcl-1 is often a primary mode of both acquired and intrinsic resistance to therapy with venetoclax^13,56^. However, the baseline features that make patients less likely to respond to venetoclax-based regimens are not completely understood. Recently, it has been shown that AML with monocytic differentiation characteristics are resistant to venetoclax‐based regimens. Moreover, following prolonged administration of the drug, monocytic clones of leukemic cells emerge, which highly express Mcl‐1 and preferentially rely on Mcl-1 for survival^57^. Intriguingly, differentiated monocytic cells, abundantly present in FAB M4 and M5 subtypes, express the highest CD157 levels compared to all other less differentiated AML subtypes^26^. It is tempting to envision that high levels of CD157 expression could be a useful tool for the prospective identification of patients who are unlikely to respond to venetoclax, but most likely to benefit from Mcl-1 inhibition. It is reasonable to presume that in addition to CD157 expression levels, a more refined strategy will include other molecular features such as genetic mutations. So far, no correlation has emerged between CD157 expression and specific mutations in a small cohort of molecularly heterogeneous AML patients analyzed^25^. However, additional studies will require a much larger cohort of AML patients before conclusions can be drawn.

## Conclusions

Increasing evidence supports the view that targeting apoptotic mechanisms is an effective strategy for treating AML^58^. Finding optimal combinations of novel targeted agents, with minimal toxicity and maximal synergy, will be a critical task to deal with in the future to better address the dynamic complexity of AML and improve patient survival. Likewise, identifying predictive biomarkers for selection of patients most likely to benefit from specific anti-apoptotic combination regimens is essential for optimal design of therapies. Overall, the results of this study suggest that CD157 may be a promising candidate as a predictive marker of response to therapies exploiting Mcl-1 pharmacological inhibition.

Mcl-1 expression is essential for survival of human stem and progenitor cells and cardiac myocyte functioning, hinting to a relevant risk of severe on-target side effects in patients treated with Mcl-1 inhibitors. Clinical data gathered from ongoing phase I clinical trials (NCT02979366, NCT03672695) will provide better insight both into the therapeutic efficacy and side effects of Mcl-1 inhibitors^40^. An intriguing possibility to deliver Mcl-1 inhibitory drugs preferentially (therefore, more safely) to leukemic cells could be to conjugate them to target-directing antibodies. It is conceivable to envision that CD157-specific antibodies might be suitable candidates for this purpose.

## Supplementary Information

Supplementary Information 1.

Supplementary Information 2.

## Data Availability

All data generated or analysed during this study are included in this manuscript and in Supporting information files. Part of data included in Supporting information are available at https://doi.org/10.5281/Zenodo.4776308.

## Abbreviations

AML: Acute myeloid leukemia
AraC: Cytarabine
BM: Bone marrow
ECM: Extracellular matrix
Mcl-1: Myeloid cell leukemia-1
FCS: Fetal calf serum
mAb: Monoclonal antibody
PI: Propidium Iodide
GSK-3β: Glycogen synthase kinase 3
MFI: Mean fluorescence intensity
EC_50_: Half maximal effective concentration
MAPK: Mitogen-activated protein kinase
ERK: Extracellular signal kinase
