# Supplementary material for: CD157 signaling promotes survival of acute myeloid leukemia cells and modulates sensitivity to cytarabine through regulation of anti-apoptotic Mcl-1
Source: Sci Rep. 2021 Oct 27;11:21230. doi: 10.1038/s41598-021-00733-5 (PMC8551154; doi:10.1038/s41598-021-00733-5)
Supplement: Supplementary file 1 — Supplementary Information 1. [file 41598_2021_733_MOESM1_ESM.pdf]

CD157 signaling promotes survival of acute myeloid leukemia cells and modulates sensitivity to cytarabine through regulation of anti-apoptotic Mcl-1

Yuliya Yakymiv[1,#], Stefania Augeri[1,#], Cristiano Bracci[1], Sara Marchisio[1], Semra Aydin[2], Stefano D'Ardia[3], Massimo Massaia[4], Enza Ferrero[1], Erika Ortolan[1,*] and Ada Funaro[1,*]

[1]Laboratory of Immunogenetics, Department of Medical Sciences, University of Torino, Italy
[2]Department of Oncology, Hematology, Immuno-Oncology and Rheumatology, University of Bonn, Germany
[3]Department of Oncology, Division of Hematology, Presidio Molinette, AOU Città della Salute e della Scienza, Torino, Italy
[4]SC Ematologia, AO S. Croce e Carle, Cuneo, Italy
[#]These authors contributed equally to this work

Table S1. Clinical and biological features of AML patients

Case #	Age	Sex	FAB classification	WHO classification	FLT3 ITD	NPM1 mut	Type of sample	Blasts (%)	Blasts $CD157^{low}$ (%)	Blasts $CD157^{dim}$ (%)	Blasts $CD157^{high}$ (%)
1	57	F	M5b	Acute monocytic leukemia	-	+	PB	44	4	40	
2	50	M	M4	Acute myelomonocytic leukemia	+	-	BM	92		92	
3	38	M	M4	Acute myelomonocytic leukemia	-	-	BM	44		29	15
4	51	F	M4	Acute myelomonocytic leukemia	+	+	PB	93		93	
5	37	F	M4	Acute myelomonocytic leukemia	-	-	BM	85	32		53
6	52	F	NA	AML with recurrent genetic alterations	-	-	BM	43	34	9	
7	25	M	M4	Acute myelomonocytic leukemia	-	-	PB	36		27	9
8	50	F	M4	Acute myelomonocytic leukemia	+	+	BM	64		38	26
9	66	F	NA	AML with MDS	-	-	BM	53	53		
10	77	F	M4	Acute myelomonocytic leukemia	-	-	BM	85		85	
11	72	F	NA	NA	NA	NA	BM	79	55	24	
12	72	F	M2	AML with maturation	-	+	PB	70	70		
13	55	M	M1	AML with minimal maturation	-	-	PB	78	78		
14	68	F	M5	Acute monocytic leukemia	-	+	PB	47			47
15	47	F	M2	AML with maturation	-	-	PB	20	20		
16	77	F	M4	Acute myelomonocytic leukemia	-	-	PB	23	2		21
17	53	F	M4	Acute myelomonocytic leukemia	-	-	PB	76	10		66
18	69	F	M2	AML with maturation	+	+	PB	77	22	55	
19	86	M	NA	Secondary AML	-	-	PB	97	61	36	

FAB = French–American–British classification; WHO = World Health Organization classification; F = female; M = male; MDS = myelodysplastic syndrome; FLT3 = fms-like tyrosine kinase 3 gene; ITD = internal tandem duplication; NPM1 = nucleophosmin 1 gene; mut = mutated; NA = not available; PB = peripheral blood; BM = bone marrow

CD157 Mean Fluorescence Intensity (MFI) ratio was calculated according to the formula (MFI of CD157 on blasts - MFI of CD157 on lymphocytes) / MFI of CD157 on lymphocytes.
CD157^{low} = MFI ratio <10; CD157^{dim} = MFI ratio ≥10 <50; CD157^{high} = MFI ratio ≥50

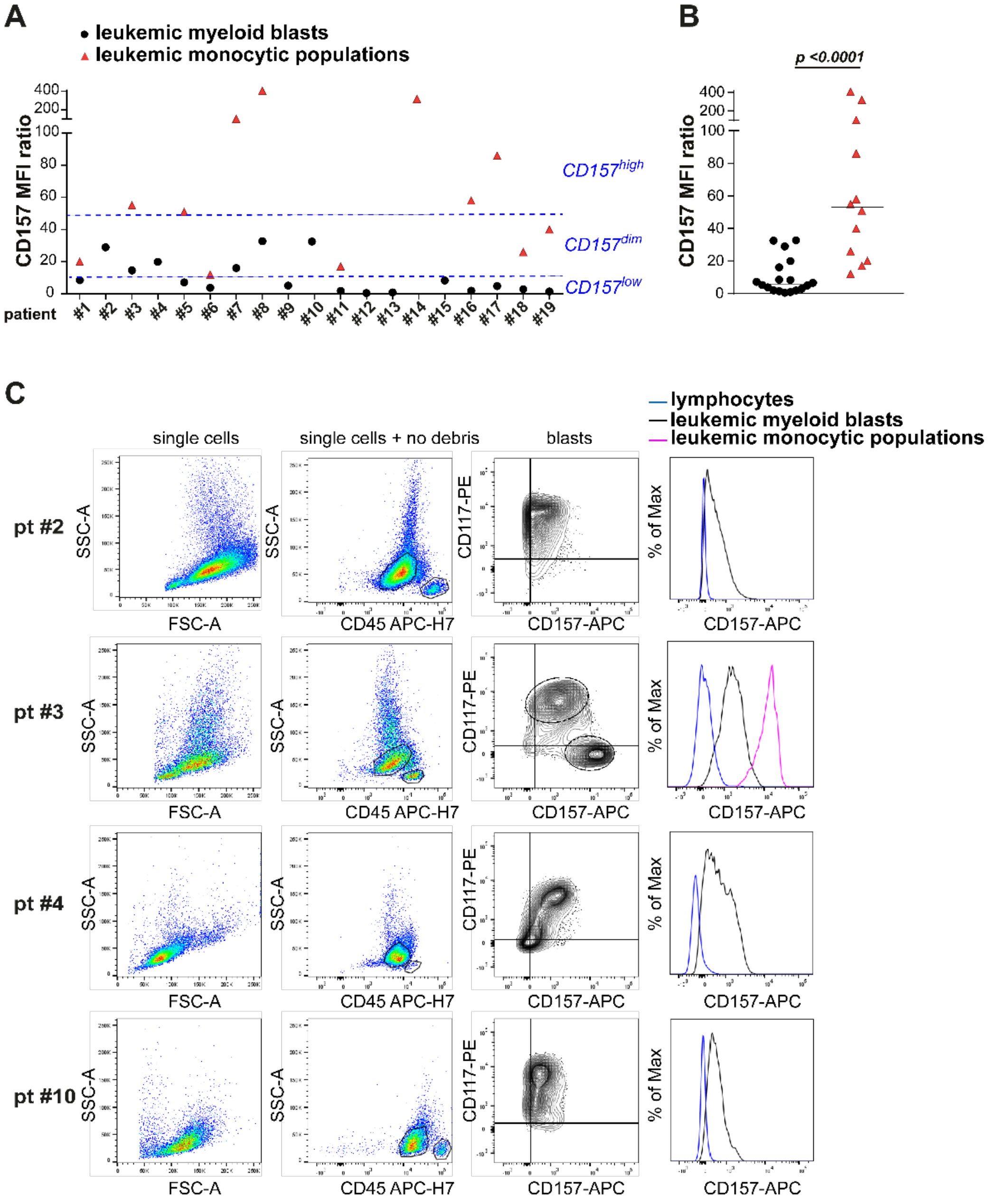

Figure S1. CD157 expression in primary AML cells assessed by multiparametric flow cytometry analysis. Leukemic blasts were identified as CD45^{dim} and SSC^{low}; specifically, myeloid blasts were CD33^{dim}, CD64-, CD117+, CD123+, HLA-DR+. Immature monocytic populations were CD33+, CD64++, CD117$^{low/neg}$, CD123+, HLA-DR++. CD157-MFI ratio was calculated according to the formula: (CD157 MFI on blasts – CD157 MFI on lymphocytes)/CD157 MFI on lymphocytes. (**A**) CD157 expression on leukemic myeloid blasts and leukemic monocytic populations in each patient. Blue dotted lines indicate the CD157 MFI ratio used as cutoff: CD157^{low} = MFI ratio <10; CD157^{dim} = MFI ratio ≥10 <50; CD157^{high} = MFI ratio ≥50. (**B**) Comparison of CD157 expression in leukemic myeloid blasts versus leukemic monocytic populations. CD157 expression was significantly higher on leukemic monocytic populations than on leukemic myeloid blasts (p <0.0001, Mann-Whitney test). (**C**) Representative FACS plots of CD157 expression in bone marrow (pt #2, pt #3 and pt #10) or peripheral blood (pt #4) from patients with AML used for functional experiments showed in Fig. 1. After excluding doublets and debris, blasts and lymphocytes were selected based

on their SSC and CD45 expression profiles. CD117 expression was used to further characterize blast populations.

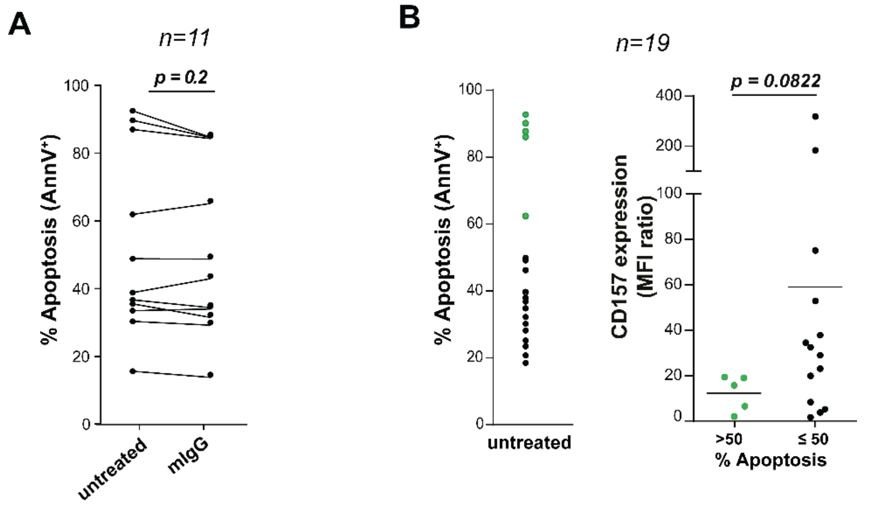

Figure S2. Impact of CD157 expression levels on AML cell survival *ex vivo*. (**A**) Mononuclear cells from AML patients (n = 11) were maintained for 24 h under standard culture conditions (untreated) or in the presence of mIgG (10 µg/ml), then the percentage of apoptotic cells was determined by flow cytometry analysis ($p = 0.02$, Wilcoxon's signed-rank test). (**B**) Mononuclear cells from AML patients (n = 19) were maintained for 24 h under standard culture conditions (untreated), then the percentage of apoptotic cells was determined by Annexin V/PI staining and flow cytometry analysis (left panel). Each dot represents a single patient. Green dots represent samples with apoptotic cells >50%; black dots represent samples with apoptotic cells ≤50%. Right panel depicts CD157 expression levels in AML cells with high and low spontaneous apoptosis *ex vivo*. (p = 0.0822, Mann-Whitney test).

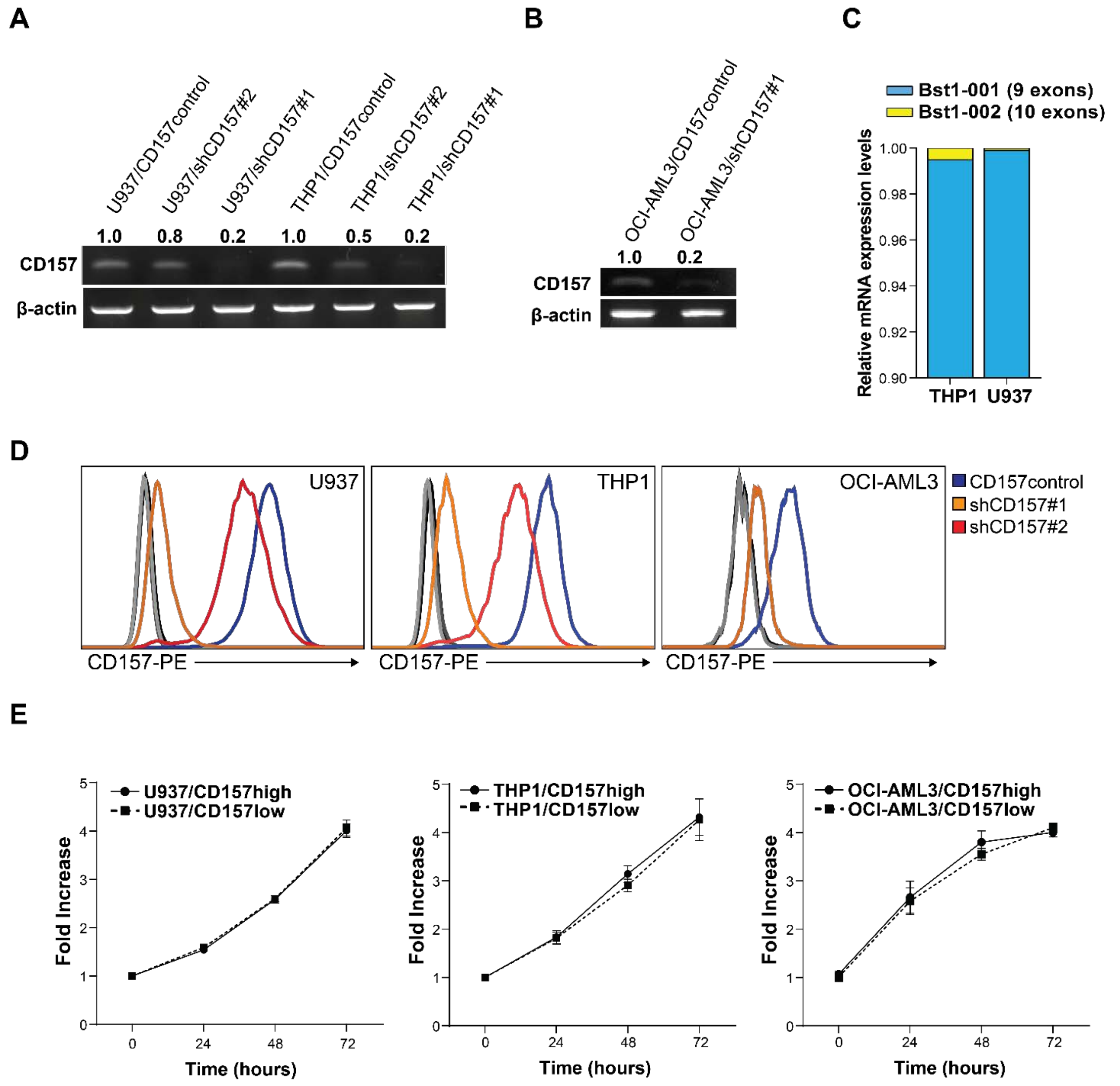

Figure S3. CD157 expression in AML cell lines. (**A**) U937 and THP1 cells were stably transduced with two CD157-specific shRNA (shCD157#1, shCD157#2) or a scrambled shRNA (CD157control). (**B**) OCI-AML3 cells were stably transduced with CD157-specific shRNA#1. CD157 mRNA (**A-B**) were analysed by RT-PCR. β-actin was used as controls, respectively. Uncropped gel images are provided in Supplementary Fig. S13. (**C**) Relative mRNA levels of *BST1-001* and *BST1-002* transcripts were determined by RTqPCR expression analysis, as described in[1]. The same cDNA amount of *BST1-001* and *BST1-002* was added to both assays. The threshold of *GUSB* was used to determine the ratio of each transcript. The relative expression of each transcript was calculated as the percentage of the sum of *BST1-001* + *BST1-002*. Bars represent *BST1-001* versus *BST1-002* ratio, expressed as mean of three independent experiments. (**D**) CD157 protein expression was analysed by flow cytometry. An isotype matched mAb (gray peak) was used as control. (**E**) Proliferation of U937, THP1 and OCI-AML3 AML cell lines engineered for the expression of CD157. Results are expressed as fold increase compared to time zero and are the mean ± SEM of three independent experiments performed in quadruplicate.

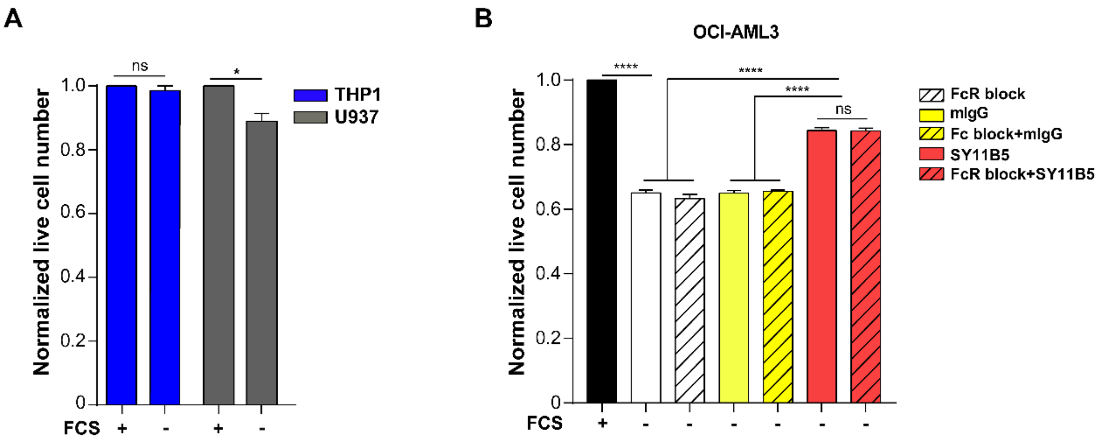

Figure S4. Effect of serum starvation on AML cell viability. (**A**) THP1 and U937 cells were cultured in the absence of FCS for 24 h (*p <0.05, Student's t-test). (**B**) OCI-AML3 cells were cultured overnight in the absence of FCS, then the SY11B5 anti-CD157 mAb or mIgG (both at 10 µg/ml) were added for 24 h in the presence or absence of mIgG Fc fragments (100 µg/ml). Cell viability was measured by AnnexinV/PI staining and flow cytometry analysis. Histograms show the effect of serum withdrawal (white bar) on cell viability expressed as fold change of viable cells compared to cells maintained in standard culture conditions (black bar), and are the mean ± SEM of three independent experiments (****p <0.0001, ns = not significant, one-way ANOVA with Tukey's multiple comparison test).

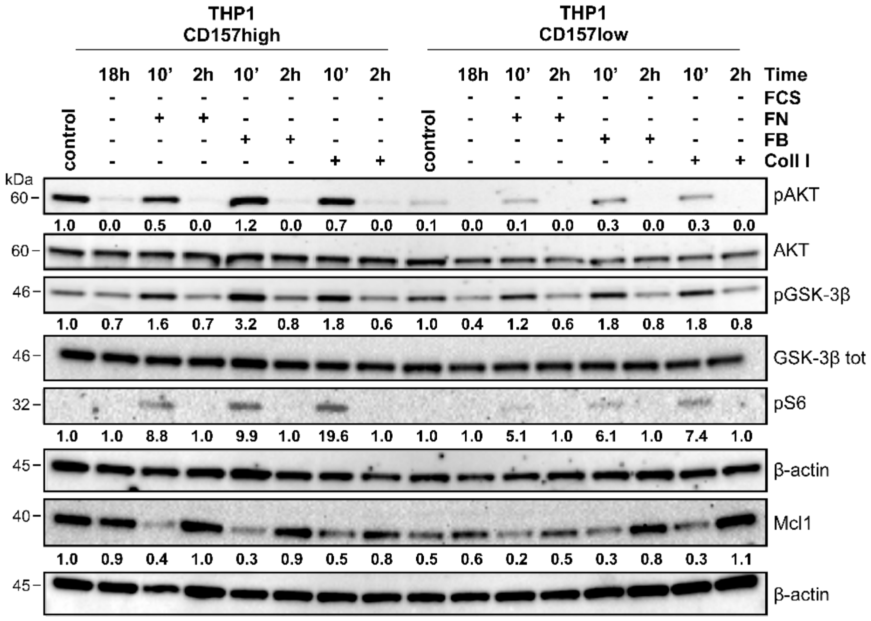

Figure S5. CD157 modulates cellular stress responses in AML cell lines. CD157-high and CD157-low THP1 cells were maintained for 18 h in FCS-free culture medium, then were stimulated with fibronectin (FN), fibrinogen (FB) or collagen type 1 (Coll I) (all at 20 µg/ml) for 10 min or 2 h. Whole cell lysates were subjected to Western blotting and probed with the indicated antibodies. Blots were re-probed using antibodies against the total proteins. β-actin was used as loading control. Numbers below blots indicate fold change in the expression of each protein relative to untreated CD157-high cells, normalized to the corresponding β-actin and total protein (AKT and GSK3β). Uncropped blot images are presented in Supplementary Fig. S14

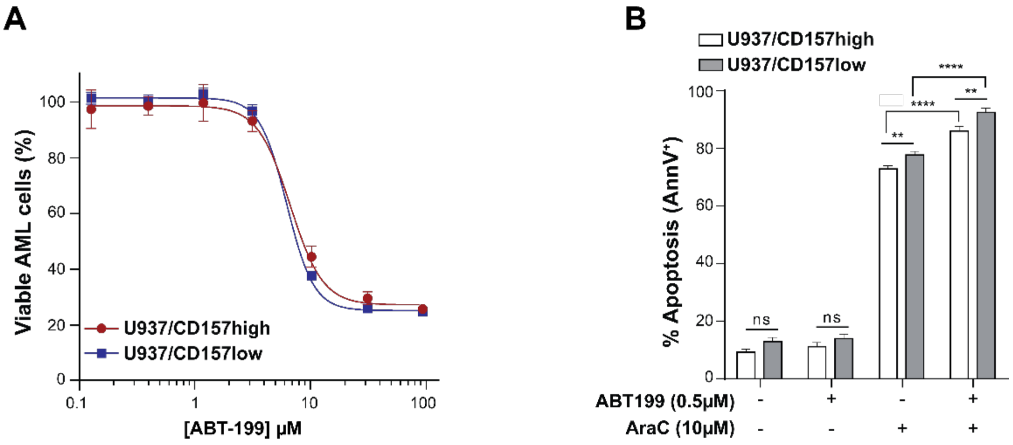

Figure S6. Effect of ABT199 on U937 cell viability. (**A**) CD157-high (red line) or CD157-low (blue line) U937 cells were exposed to increasing concentration of ABT-199 for 24 h before analysis of cell viability using Presto Blue assays. Data represents the percentage of live cells compared to control cells treated with vehicle. Results are the mean ± SEM of three experiments performed in triplicate. (**B**) CD157-high or low U937 cells were treated with vehicle or with AraC (10 µM) and ABT199 (0.5 µM) as single agents or in combination for 24 h, then were subjected to AnnexinV/PI staining and flow cytometry analysis. Bars represent the percentage of AnnexinV-positive cells and are presented as mean ± SEM, $n = 3$. (**p <0.01, ****p <0.0001, ns = not significant, two-way ANOVA with Tukey's multiple comparison test).

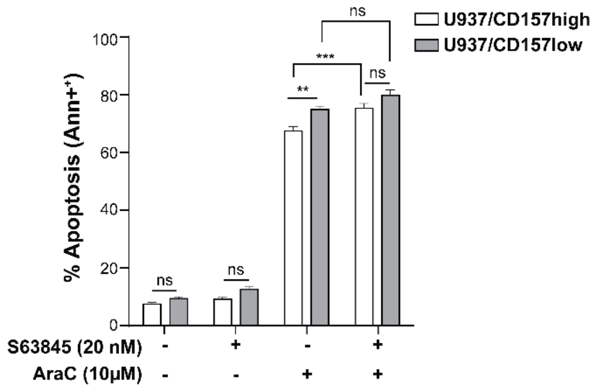

Figure S7. Effect of S63845 and AraC combination on U937 cell viability. U937/CD157-high or U937/CD157-low cells were treated with AraC (10 µM) and S63845 (20 nM) as single agents or in combination for 24 h, then were subjected to AnnexinV/PI staining and flow cytometry analysis. Bars represent the percentage of apoptotic cells and are presented as mean ± SEM, $n = 11$. (**p <0.01, ***p <0.001, ns = not significant, two-way ANOVA with Tukey's multiple comparison test.

References

1 Ferrero, E. *et al.* Human canonical CD157/Bst1 is an alternatively spliced isoform masking a previously unidentified primate-specific exon included in a novel transcript. *Sci Rep.* **7**, 15923, doi:10.1038/s41598-017-16184-w (2017).
