# Supplementary material for: CD157 signaling promotes survival of acute myeloid leukemia cells and modulates sensitivity to cytarabine through regulation of anti-apoptotic Mcl-1
Source: Sci Rep. 2021 Oct 27;11:21230. doi: 10.1038/s41598-021-00733-5 (PMC8551154; doi:10.1038/s41598-021-00733-5)
Supplement: Supplementary file 2 — Supplementary Information 2. [file 41598_2021_733_MOESM2_ESM.pdf]

CD157 signaling promotes survival of acute myeloid leukemia cells through regulation of Mcl-1

Yuliya Yakymiv[1,#], Stefania Augeri[1,#], Cristiano Bracci[1], Sara Marchisio[1], Semra Aydin[2], Stefano D'Ardia[3], Massimo Massaia[4], Enza Ferrero[1], Erika Ortolan[1,*] and Ada Funaro[1,*]

[1]Laboratory of Immunogenetics, Department of Medical Sciences, University of Torino, Italy
[2]Department of Oncology, Hematology, Immuno-Oncology and Rheumatology, University of Bonn, Germany
[3]Department of Oncology, Division of Hematology, Presidio Molinette, AOU Città della Salute e della Scienza, Torino, Italy
[4]SC Ematologia, AO S. Croce e Carle, Cuneo, Italy
[#]These authors contributed equally to this work

Uncropped blot images

Supplementary Fig. S8

Figure 1F/1G

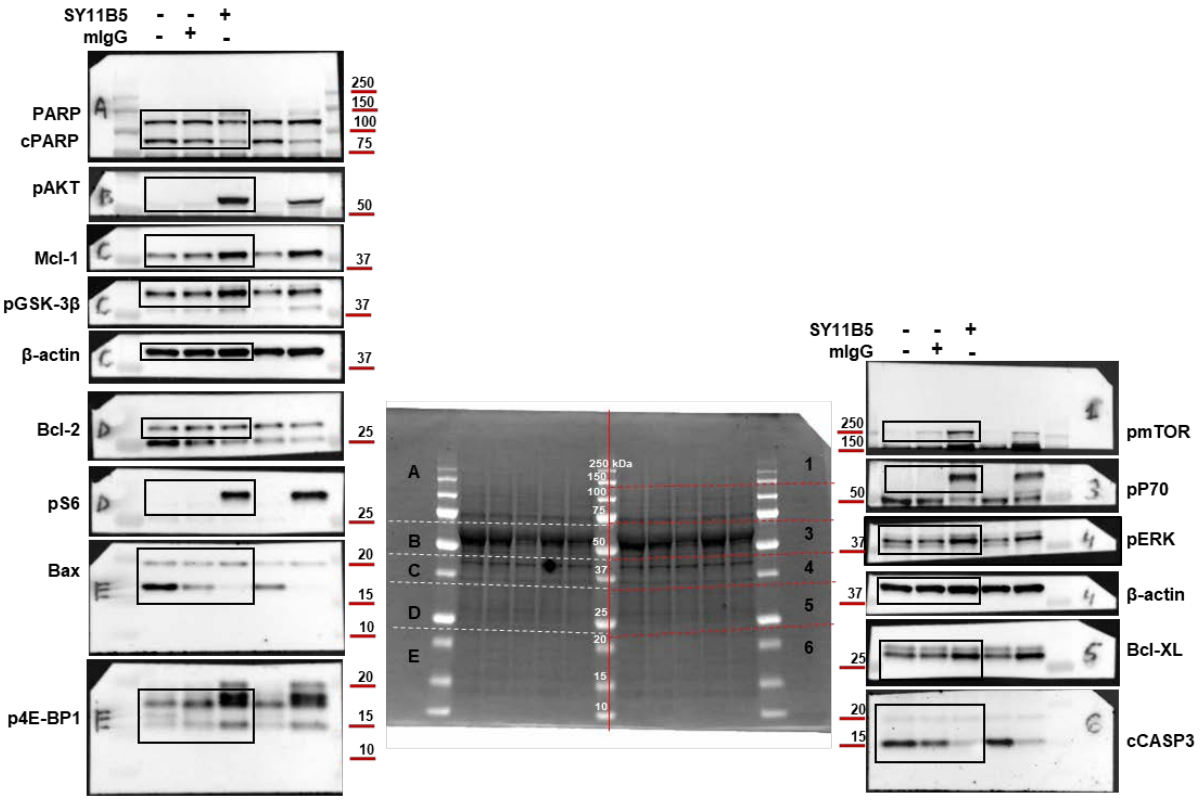

membrane	***1st probe***	***2nd probe***	***3rd probe***
A	PARP		
B	pAKT		
C	Mcl-1	pGSK-3β	β-actin-HRP (Fig. 1F)
D	Bcl-2	pS6	
E	Bax	p4E-BP1	

membrane	***1st probe***	***2nd probe***
1	pmTOR	
3		pP70
4	pERK	β-actin-HRP (Fig. 1G)
5	Bcl-XL	
6	cCASP3	

Uncropped blot from western blot analysis presented in Figure 1F-G. Panel F and G were generated from the same blot using the same samples. Dotted lines in full-length blot indicate the excision lines. Each strip was separately probed with the indicated antibodies. After hybridization with the first probe, the membrane was stripped and re-hybridized with the second probe according to the table under each blot. The rectangles show the cropped region of the blots included in Fig. 1F-G.

Supplementary Fig. S9

Figure 2B

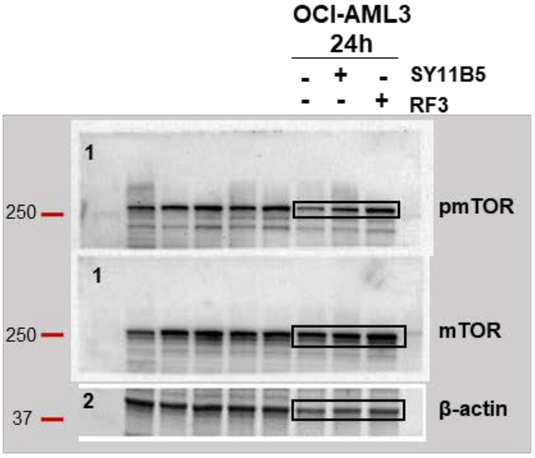

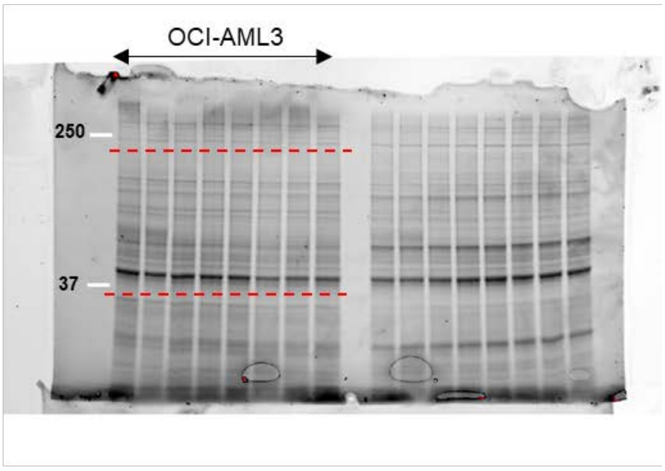

membrane	1st probe	2nd probe
1	pmTOR	mTOR
2		β-actin-HRP

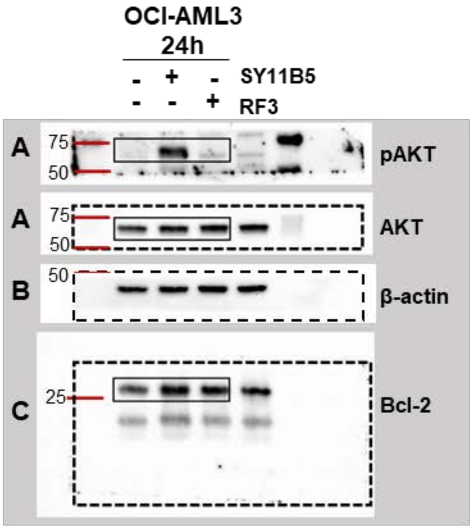

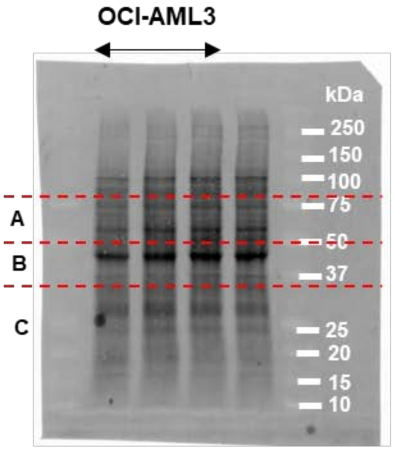

membrane	1st probe	2nd probe
A	pAKT	AKT
B	β-actin-HRP	
C	Bcl-2	

Uncropped gel (top) and blot (bottom) from western blot analysis presented in Figure 2B. Two separate blots were generated using the same samples. Dotted lines in uncropped images indicate the excision lines. Each strip was separately probed with the indicated antibodies. After hybridization with first probe, the membrane was stripped and re-hybridized with the second probe according to the table under each blot. The rectangles show the cropped region of the blots included in the figure. Black dotted lines indicate membrane periphery.

Supplementary Fig. S9 (continued)

Figure 2D

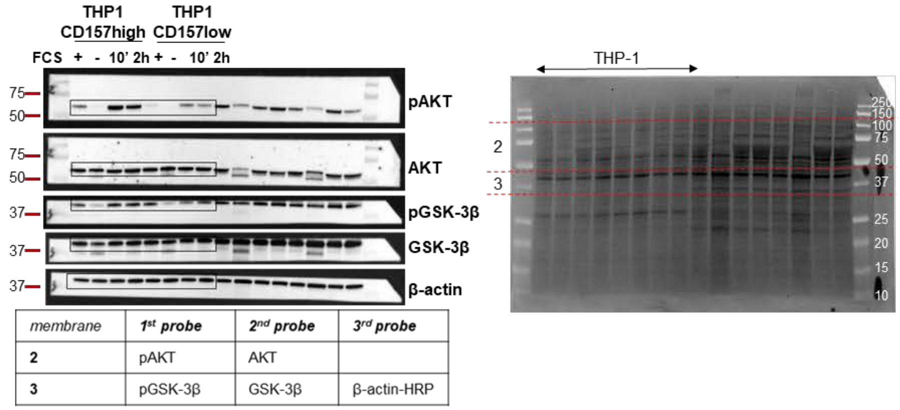

membrane	1st probe	2nd probe	3rd probe
2	pAKT	AKT	
3	pGSK-3β	GSK-3β	β-actin-HRP

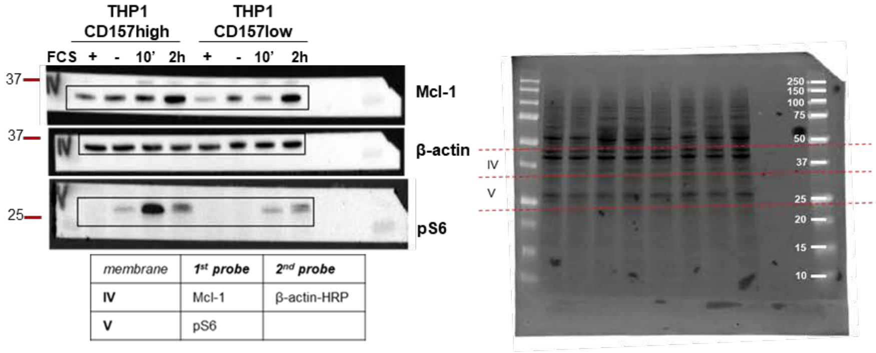

membrane	1st probe	2nd probe
IV	Mcl-1	β-actin-HRP
V	pS6	

Figure 2E

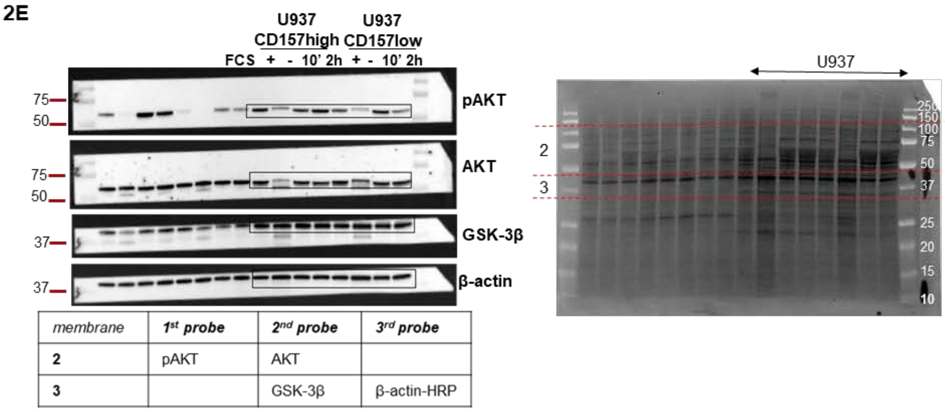

membrane	1st probe	2nd probe	3rd probe
2	pAKT	AKT	
3		GSK-3β	β-actin-HRP

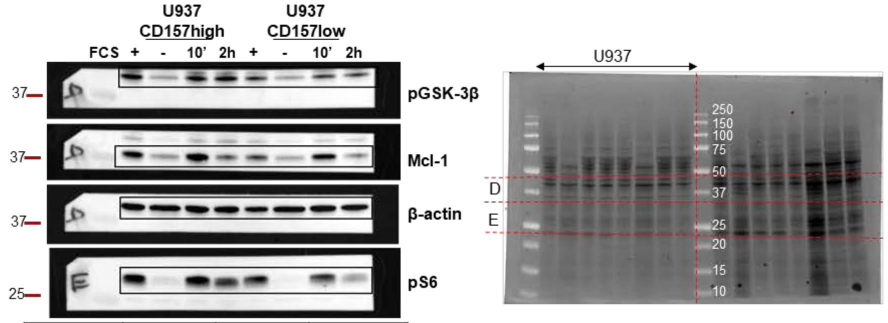

membrane	1st probe	2nd probe	3rd probe
D	pGSK-3β	Mcl-1	β-actin-HRP
E	pS6		

Uncropped blots from western blot analysis presented in Figure 2D-E. For each cell line two separate blots were generated using the same samples. Dotted red lines in full-length blot indicate the excision lines. Each strip was separately probed with the indicated antibodies. After hybridization with first probe, the membrane was stripped and re-hybridized with the second probe according to the table under each blot. The rectangles show the cropped region of the blots included in the figure.

Supplementary Fig. S10

Figure 3D

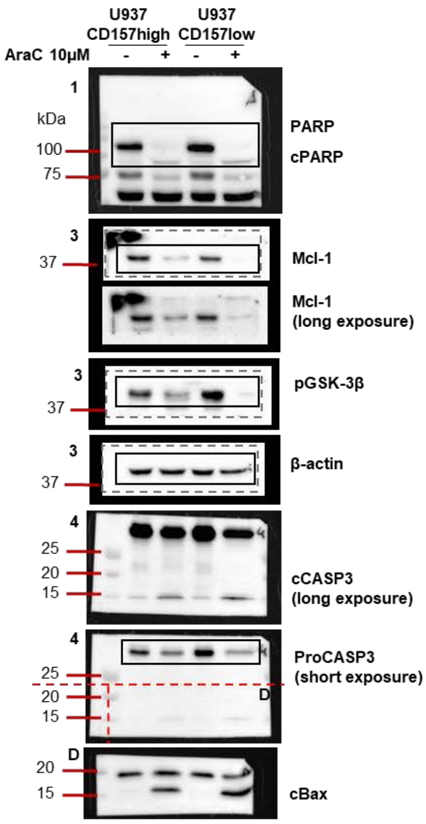

membrane	1st probe	2nd probe	3rd probe
1	PARP		
3	Mcl-1	pGSK-3β	β-actin-HRP
4	CASP3		
D		Bax	

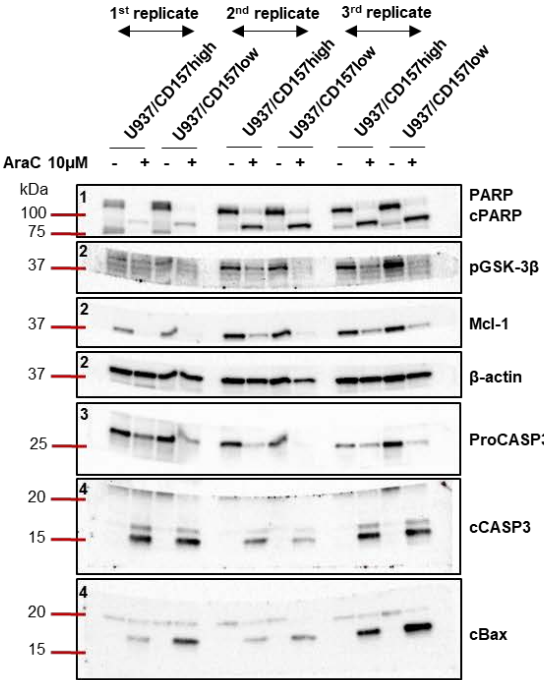

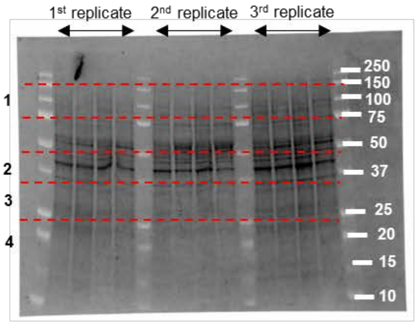

membrane	1st probe	2nd probe	3rd probe
1	PARP		
2	Mcl-1	pGSK-3β	β-actin-HRP
3	CASP3		
4	CASP3	Bax	

Uncropped blot presented in Figure 3D. Upper blot was cut prior to hybridization and each strip was separately probed with the indicated antibodies. After hybridization with first probe, the membrane was stripped and re-hybridized with the second probe according to each table. The rectangles show the cropped region of the blots included in Fig. 3D. Multiple exposures are shown for blots that were taken with a higher contrast or do not have background sufficiently dark to show the membrane periphery. Grey dotted lines indicate membrane periphery. Bottom blot show three independent experiments run in parallel and used for quantification of protein levels by densitometry analysis reported in Fig. 3D (left panel).

Supplementary Fig. S11

Figure 4B

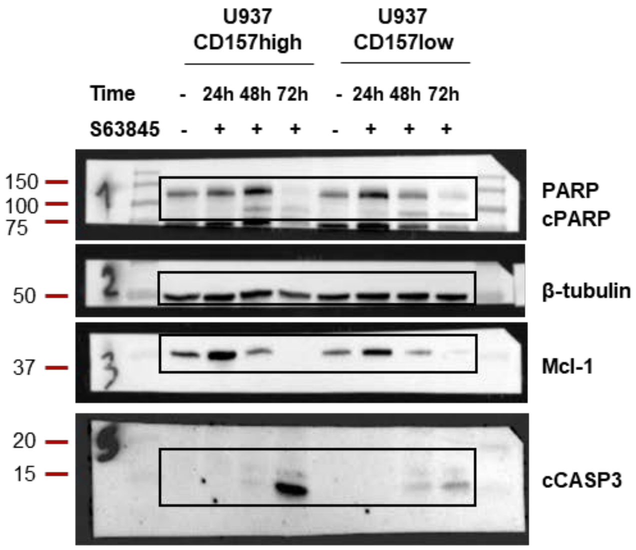

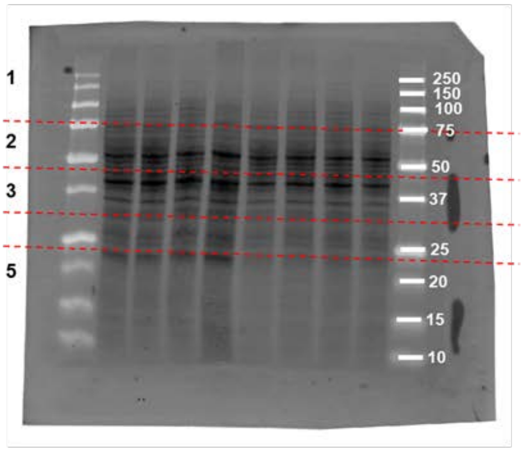

membrane	***probe***
**1**	PARP-1
**2**	β-tubulin-HRP
**3**	Mcl-1
**5**	cCASP3

Uncropped version of blot presented in Figure 4B. The rectangles show the cropped region of the blots included in the figure. Dotted red lines in full-length blot indicate the excision lines. Each strip was separately probed with the indicated antibody.

Supplementary Fig. S11 (continued)

Figure 4D

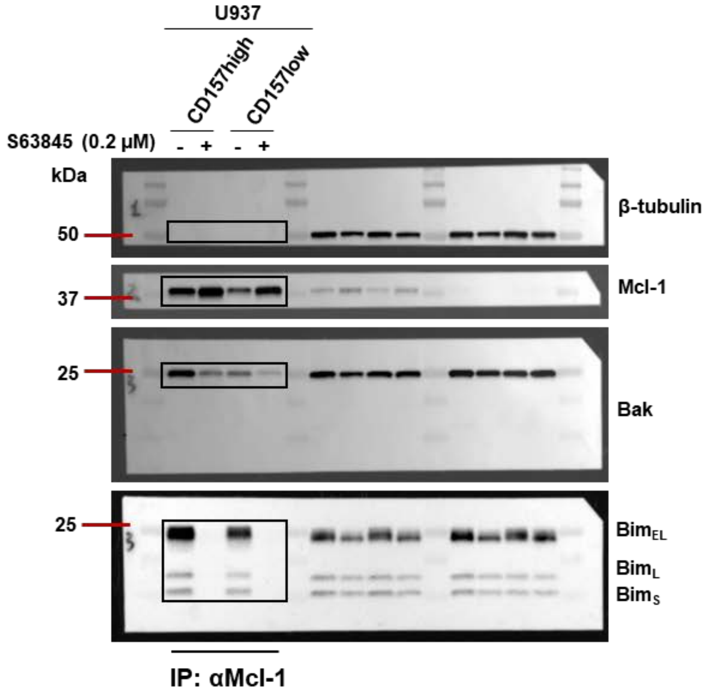

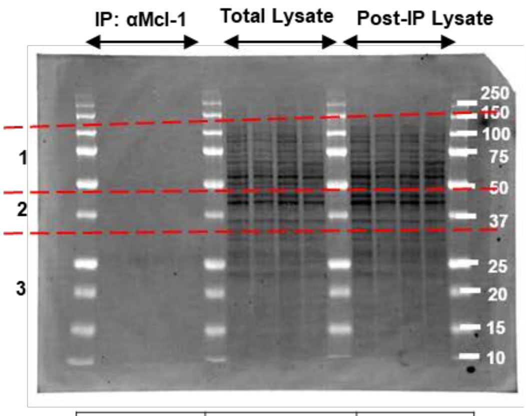

membrane	*1st probe*	*2nd probe*
1	β-tubulin-HRP	
2	Mcl-1	
3	Bak	Bim

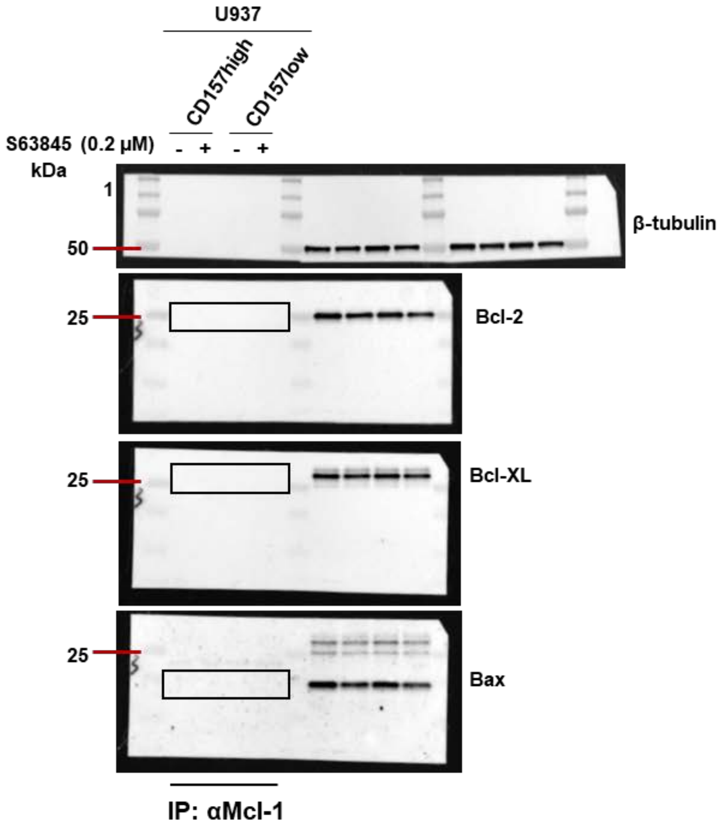

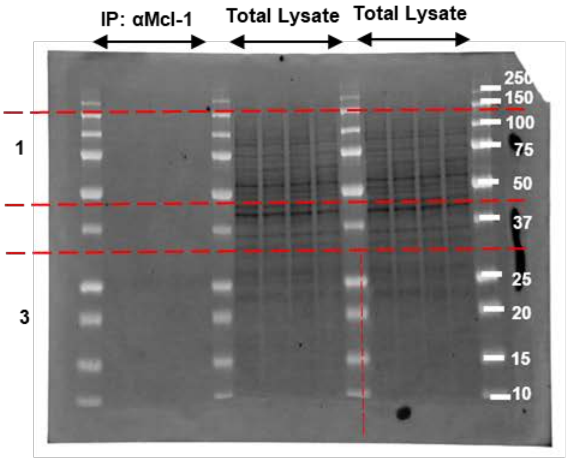

membrane	*1st probe*	*2nd probe*	*3rd probe*
1	β-tubulin-HRP		
3	Bcl-2	Bcl-XL	Bax

Supplementary Fig. S11 (continued)

Figure 4D

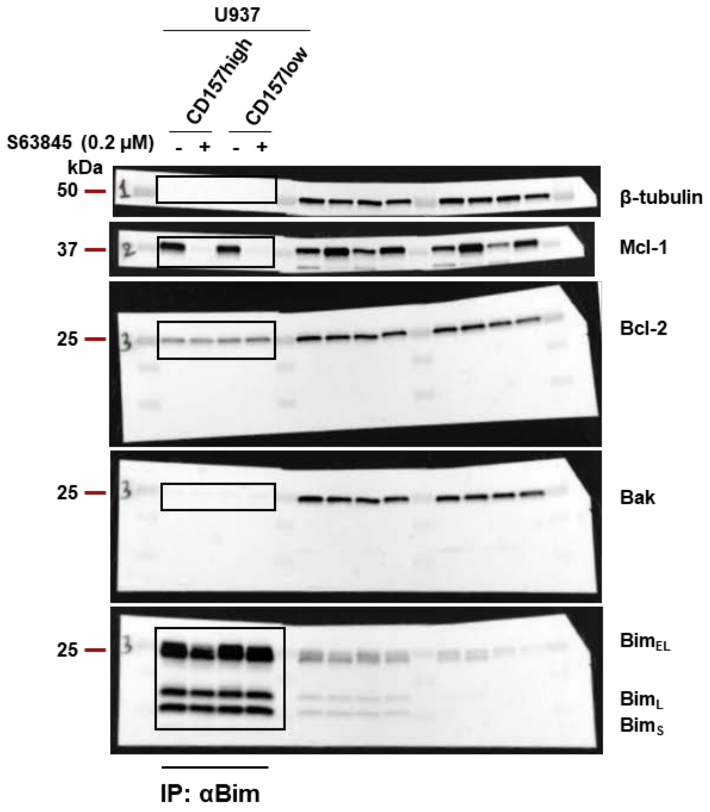

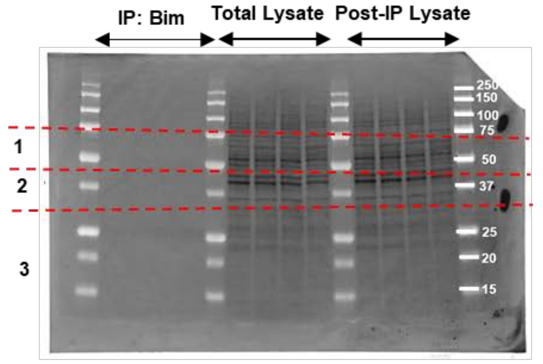

membrane	***1st probe***	***2nd probe***	***3rd probe***
1	β-tubulin-HRP		
2	Mcl-1		
3	Bcl-2	Bak	Bim

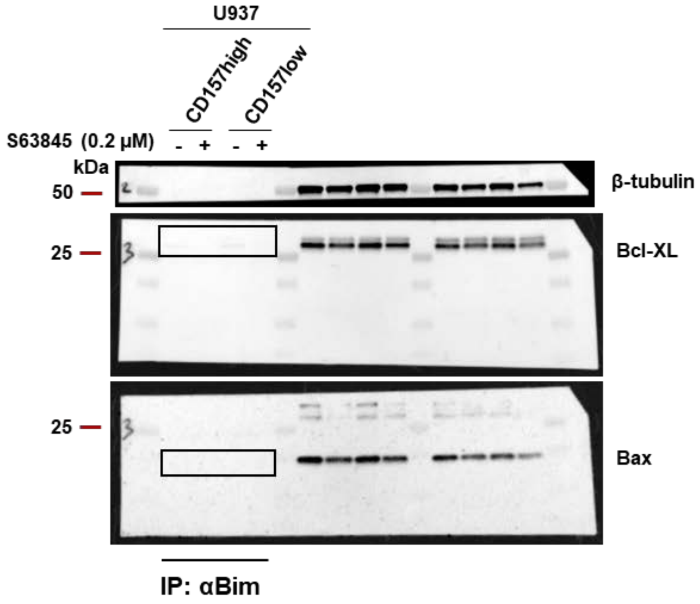

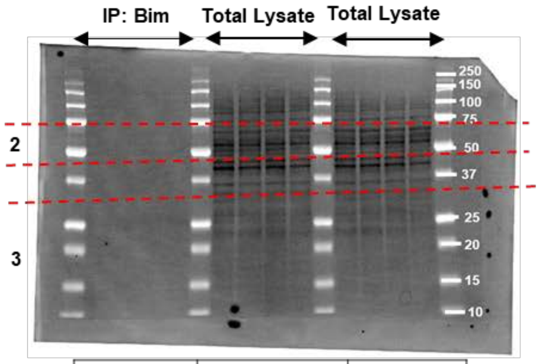

membrane	***1st probe***	***2nd probe***
2	β-tubulin-HRP	
3	Bcl-XL	Bax

Supplementary Fig. S11 (continued)

Figure 4D

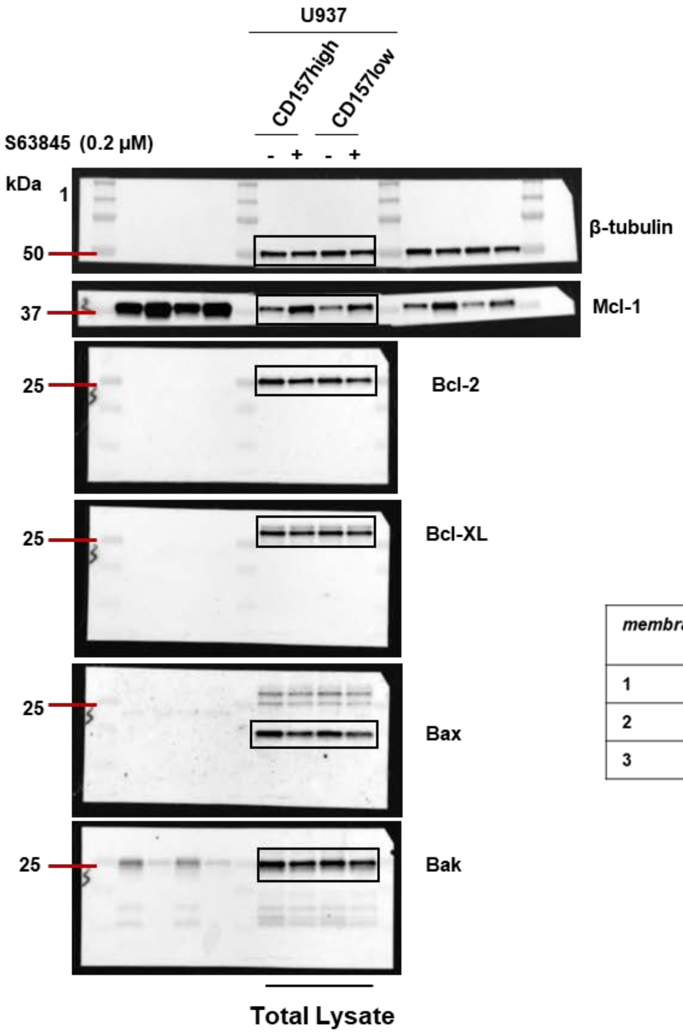

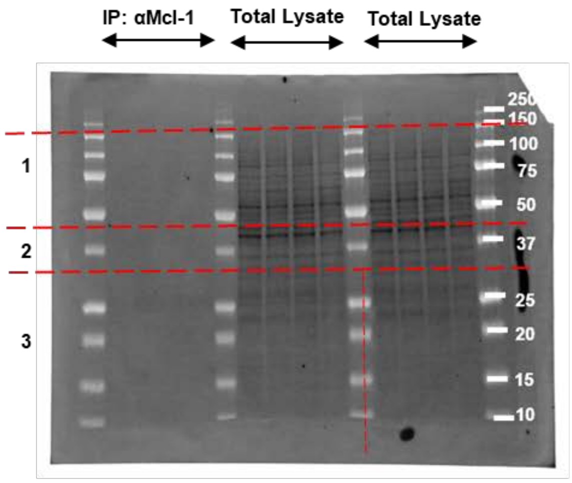

membrane	*1st probe*	*2nd probe*	*3rd probe*	*4th probe*
1	β-tubulin-HRP			
2	Mcl-1			
3	Bcl-2	Bcl-XL	Bax	Bak

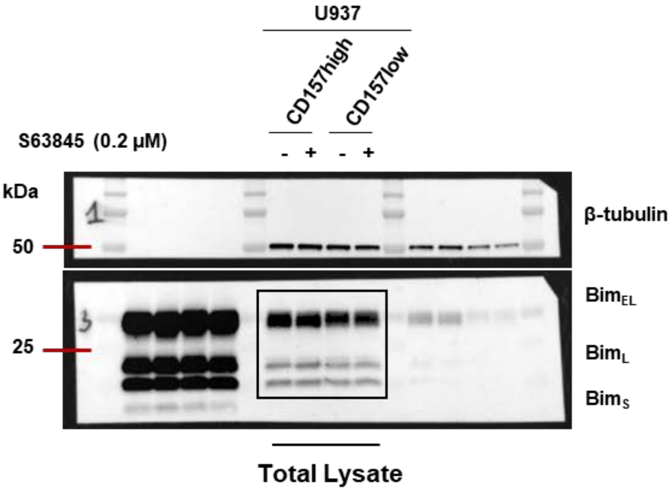

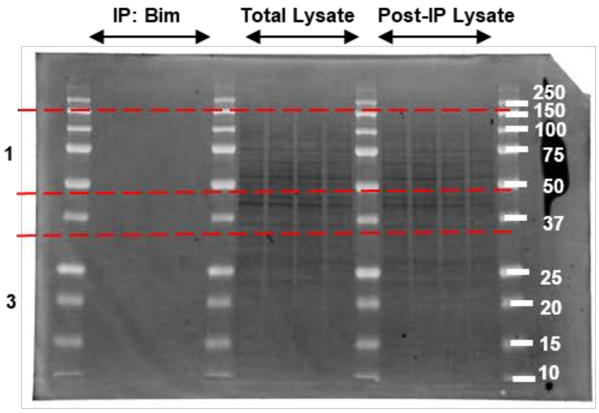

membrane	*1st probe*
1	β-tubulin-HRP
3	Bim

Uncropped blot from the western blot analysis presented in Figure 4D. All blots were generated using the same samples. Dotted red lines in uncropped images indicate the excision lines. Each strip was separately probed with the indicated antibodies. After hybridization with first probe, the membrane was stripped and re-hybridized with the second probe according to the table under each blot. The rectangles show the cropped region of the blots included in Fig. 4D.

Supplementary Fig. S12

Figure 5C

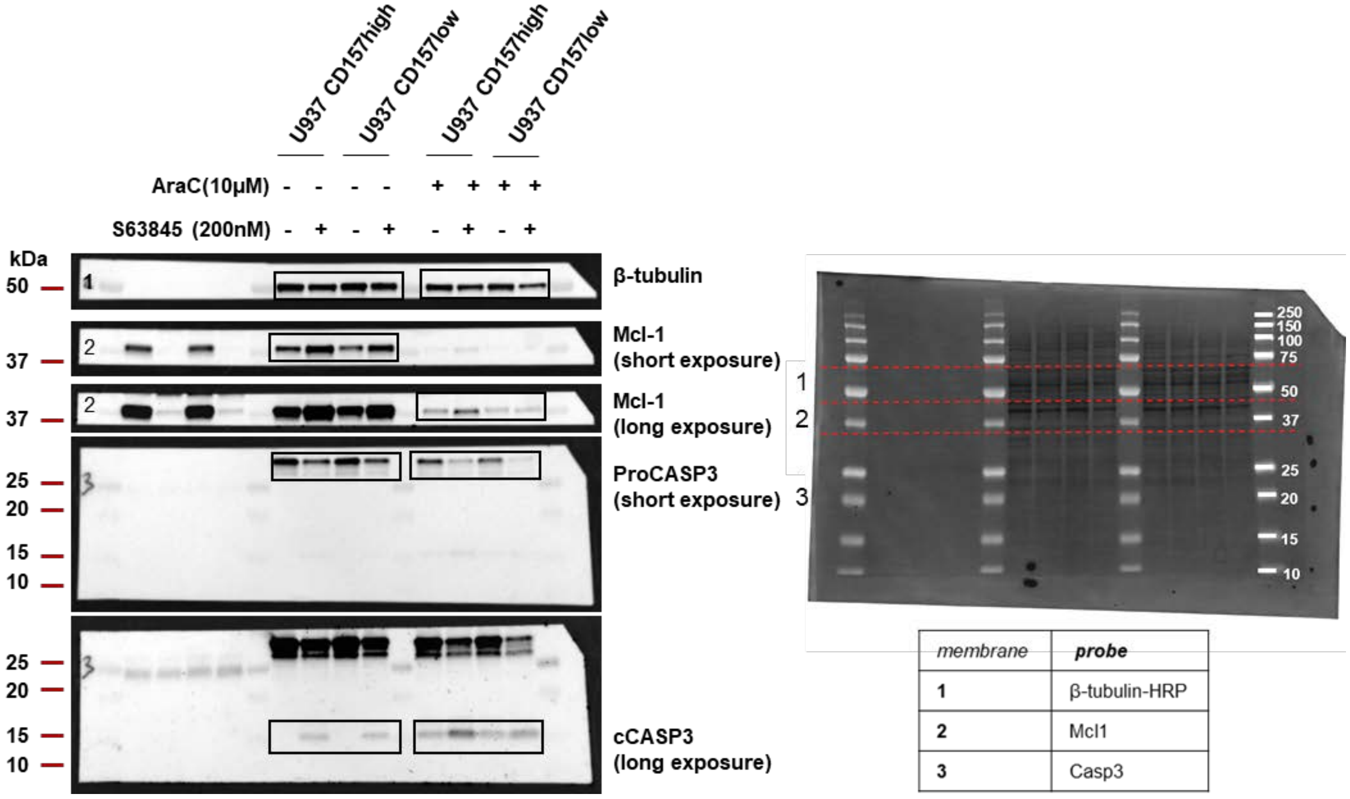

membrane	***probe***
1	β-tubulin-HRP
2	Mcl1
3	Casp3

Uncropped blot from the western blot analysis presented in Figure 5C. Dotted lines in uncropped images indicate the excision lines. Each strip was separately probed with the indicated antibody. Multiple exposures are shown for blots that were taken with a higher contrast The rectangles show the cropped region of the blots included in Fig. 5C.

Supplementary Fig. S13

Figure S3

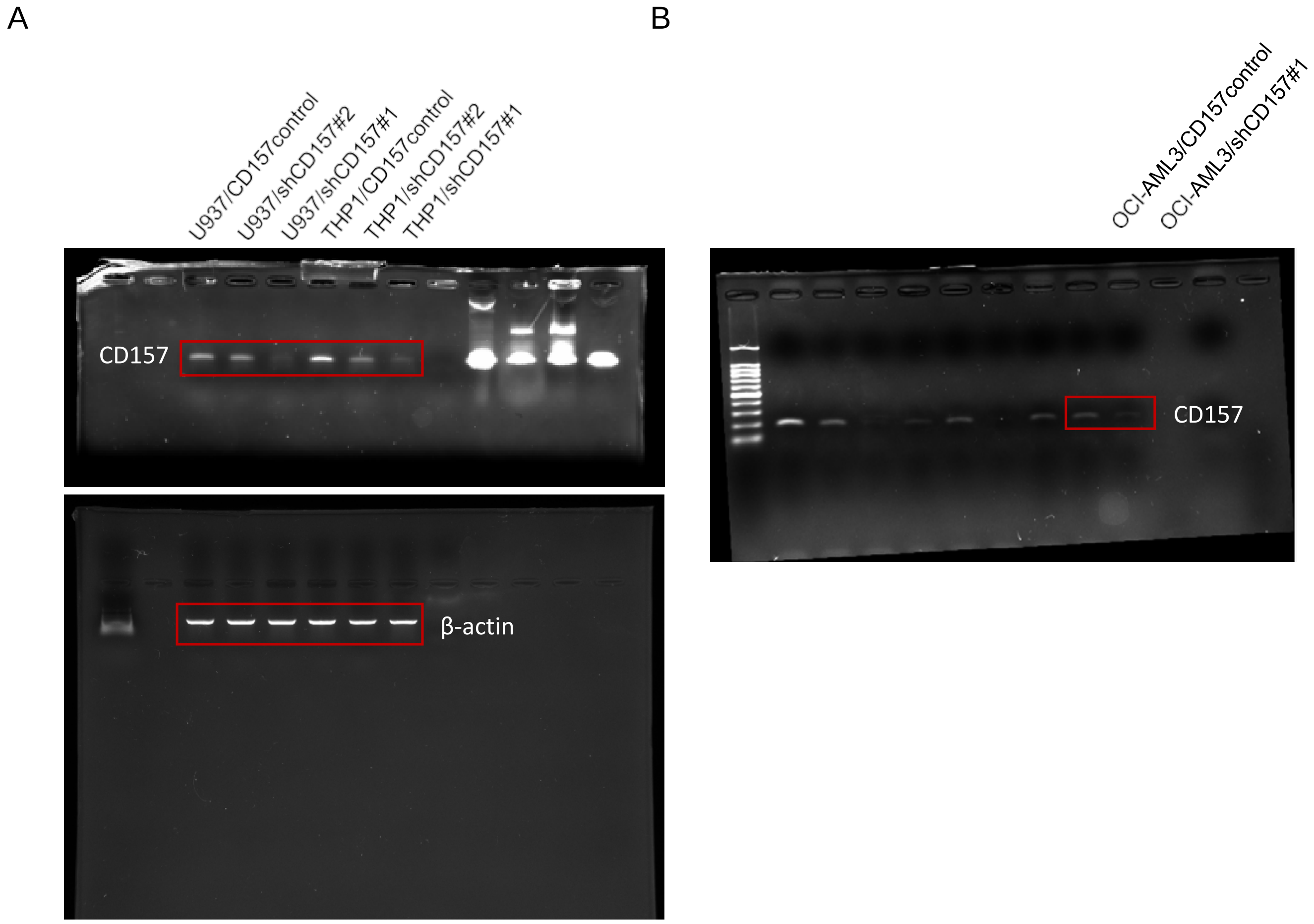

Uncropped gels from PCR analysis performed in Figure S3. The rectangles show the cropped region of the gels included in Fig. S3.

Supplementary Fig. S14

Figure S5

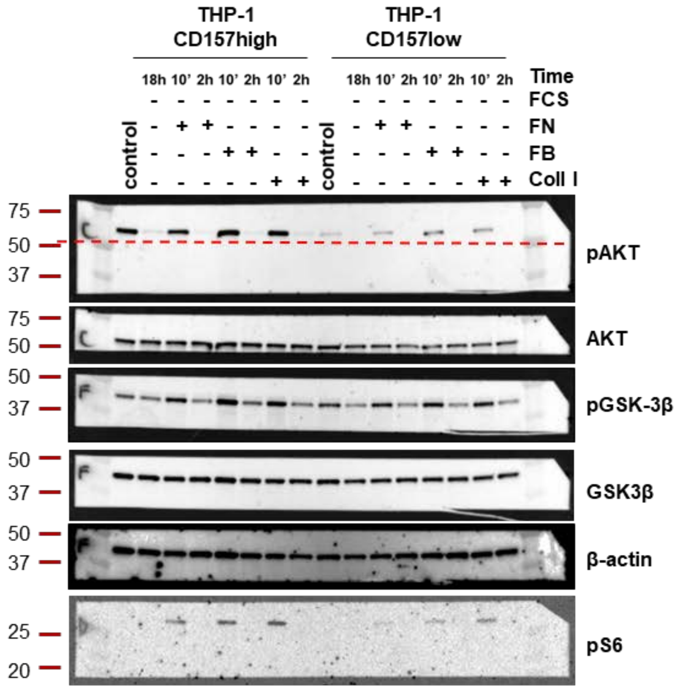

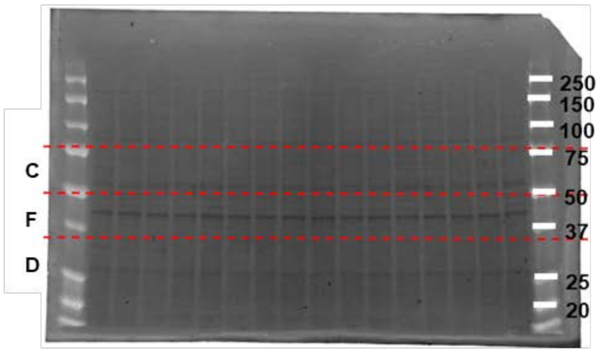

membrane	*1st probe*	*2nd probe*	*3rd probe*
C	pAKT	AKT	
F	pGSK-3β	GSK-3β	β-actin-HRP
D	pS6		

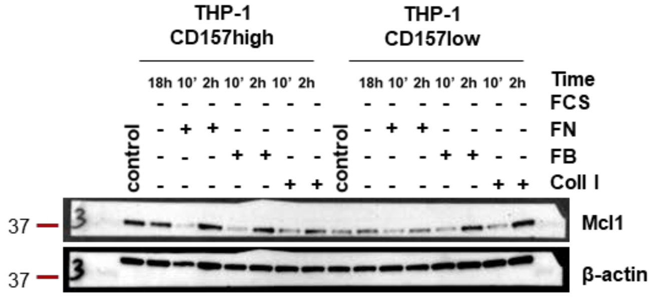

membrane	*1st probe*	*2nd probe*
3	Mcl-1	β-actin-HRP

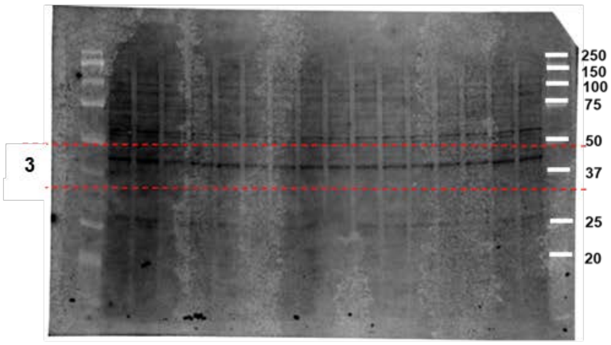

Uncropped blots from western blot analysis presented in Figure S5. Two separate blots were generated using the same samples. Dotted red lines in full-length blots indicate the excision lines. Each strip was separately probed with the indicated antibodies. After hybridization with first probe, the membrane was stripped and re-hybridized with the second probe according to the table under each blot.
